# Modeling transport of extended interacting objects with drop-off phenomenon

**DOI:** 10.1371/journal.pone.0267858

**Published:** 2022-05-02

**Authors:** Aditi Jain, Arvind Kumar Gupta

**Affiliations:** Department of Mathematics, Indian Institute of Technology Ropar, Rupnagar, Punjab, India; Southwest Jiaotong University, CHINA

## Abstract

We study a deterministic framework for important cellular transport phenomena involving a large number of interacting molecules called the *excluded flow of extended interacting objects with drop-off effect* (EFEIOD). This model incorporates many realistic features of biological transport process including the length of biological “particles” and the fact that they can detach along the biological ‘tracks’. The flow between the consecutive sites is unidirectional and is described by a “soft” simple exclusion principle and by repelling or attracting forces between neighboring particles. We show that the model admits a unique steady-state. Furthermore, if the parameters are periodic with common period *T*, then the steady-state profile converge to a unique periodic solution of period *T*. Simulations of the EFEIOD demonstrate several non-trivial effects of the interactions on the system steady-state profile. For example, detachment rates may help in increasing the steady-state flow by alleviating traffic jams that can exist due to several reasons like bottleneck rate or interactive forces between the particles. We also analyze the special case of our model, when there are no forces exerted by neighboring particles, and called it as the ribosome flow model of extended objects with drop-off effect (RFMEOD), and study the sensitivity of its steady-state to variations in the parameters.

## 1 Introduction

There are many important biological transport phenomena where the driving force for the movement of particles depends upon the constant source of energy [1]. One of the most known examples of such a system is intracellular transport carried out by motor proteins which are also known as biological molecular motors. They utilize the free energy that is produced during the chemical hydrolysis of adenosine triphosphate molecules (ATP) by converting it into mechanical work for their movement along cytoskeleton protein filaments [2]. These transport processes are modeled as the unidirectional or bidirectional flow of biological “particles” (RNA polymerases, ribosomes, motor proteins) along an ordered chain of sites (DNA, mRNA, microtubules).

Experimental investigations show that in many complex cellular processes such as mRNA translation by ribosomes or intracellular transport carried by motor proteins, the particles are larger than their step sizes and they usually function in large groups and interact with one another by binding and repelling actions based on the state of its neighboring particles [3]. Also, it has been seen that in many of these transport processes, the biological particles may get detached along the tracks. For example, kinesin-family motor proteins get detached from the microtubule after every power stroke or when their path is blocked [4, 5]. Defects in kinesin-linked transport may disrupt the functioning of nerve cells and can cause many serious diseases [6]. The neuron-wide system requires intracellular transport of cargo throughout complex neuronal morphologies and its transport malfunction is one of the indications of some neuronal diseases like Alzheimer’s [7]. Therefore, deriving mathematical models of these dynamical biological phenomena is important and crucial for understanding the collective behavior of the movement of particles and unraveling its biophysical aspects in the context of synthetic biology and biomedical applications.

These transport phenomena are usually studied using several multi-particle lattice gas models [3]. The totally asymmetric simple exclusion process (TASEP) is an important model in non-equilibrium statistical physics that has been used to model molecular motors traffic and ribosome flow during mRNA translation [8–10]. In this stochastic model, particles hop unidirectionally with a step size of one along an ordered lattice of sites. The biological particles have volume and thus cannot overtake one other, i.e. as long as a site remain covered by a particle, it is inaccessible to the other particles thus obeying the simple exclusion principle. TASEP and its various versions have been introduced to model realistic observed features of interactions, extended objects and dissociations [11–16]. Unfortunately, rigorous analysis of TASEP is non-trivial and exact solutions exist in special simplified cases like the model with the homogeneous rates. Moreover detailed TASEP-type models are analyzed via various approximations and time-consuming Monte Carlo computer simulations. Therefore, a deterministic mathematical model called ribosome flow model (RFM) obtained via a mean-field approximation of TASEP has been introduced that is both amenable to mathematical analysis using tools from systems and control theory [17, 18] and is easy to simulate. The analysis holds for any set of feasible parameter values. The RFM and its various extensions have been used to analyze many biological processes including positive feedbacks [19], the effect of competition for shared resources in translation [20], extended length of particles [29], bidirectional flow with Langmuir kinetics [21], networks of interconnected mRNAs [22], etc. Also, the analysis of the TASEP and its various extensions always provide approximate results which become accurate as number of sites *n* goes to infinity whereas analysis of the RFM and its various extensions hold true for every *n*.

An extension of the RFM called excluded flow with local repelling and binding model (EFRBM) was introduced to study the nearest-neighbor interactions between the motor proteins [23]. The advantage of this model is that it is that is amenable to rigorous analysis even in case of non-homogeneous rates. The EFRBM is a non-linear, continuous-time compartmental model for the unidirectional flow of interacting particles along a one-dimensional chain of *n* consecutive compartments. In this model, each particle is assumed to cover a single site and the nearest-neighbor effect is modeled by two interaction parameters *q* and *r*. The rate of movement of particle from site *i* to *i* + 1 depends upon the parameter *q* ≥ 0 [*r* ≥ 0] if site *i* + 2 [*i* − 1] is already occupied. A value *q* > 1 [*q* < 1] implies that the particle at site *i* will be strongly attracted [repelled] to the particle at site *i* + 2. This means that particle will move faster from site *i* to *i* + 1 creating a new bond with particle at site *i* + 2. Similarly a value *r* > 1 [*r* < 1] represents the detachment[attachment] force at site *i* by the neighboring particle at site *i* − 1. It has been shown that the trajectories of EFRBM evolve on the compact and convex set *C*^*n*^ ≔ [0, 1]^*n*^.

In this paper, we extend the EFRBM to include the fact biological “particles” cover several sites and are susceptible to detach at various sites along the lattice. We refer to this model as the *excluded flow of extended interacting objects with drop-off effect* (EFEIOD). Using the theory of contractive dynamical systems, we prove that EFEIOD always converges to a steady-state. This steady-state depends on the length of the lattice *n*, the particle size *ℓ*, transition rates λ_*i*_s, detachment rates *α*_*i*_s and the interaction parameters *q* and *r* but not on the initial conditions. We also prove that it entrains to periodic excitations in the transition/detachment rates and the interaction parameters. This is important for the proper functioning of the biological processes that are excited by the periodic events. Analysis and simulations highlight the role of the effect of interactions on the steady-state flow. For example, in the case of strong attractions from the neighboring particle at site *i* − *ℓ*, the flow of particles from site *i* to *i* + 1 gets reduced, therefore an increase in detachment rate of particles at site *i* − *ℓ* leads to an easy steady-state flow. In the absence of interactions, we analyze the transport phenomena of mRNA translation with ribosome drop-off and called it RFMEOD. We also show using simulations that the RFMEOD correlates well with the TASEP with extended objects and including the drop-off phenomenon.

The EFEIOD presented here is more general than the EFRBM as it includes biologically observed more features such as particles with extended length and phenomena of dissociation of particles along the tracks.

The remainder of the paper is organized as follows. Section 2 describes the mathematical model. The next section presents our main theoretical results and the effects of the nearest-neighbor interactions on the steady-state behavior. Section 4 describes the application of the EFEIOD to model mRNA translation with ribosome drop-off and allows us to understand how a change in one of the parameters affects protein production. The final section concludes and summarizes the paper. To increase the readability of the paper, all the proofs are placed in the Appendix.

## 2 Model

The EFEIOD is a nonlinear, continuous time, compartmental model for the unidirectional flow of biological “particles” of size *ℓ* directed from left to right on a one-dimensional chain of *n* consecutive compartments or sites along the track.

The EFEIOD contains the following sets of 2*n* + 3 non-negative parameters:

1. λ_*i*_ > 0, *i* = 0, 1, …, *n*: controls the transition rate from site *i* to *i* + 1.
2. *α*_*i*_ ≥ 0, *i* = 1, …, *n*: controls the detachment rate from site *i* to the environment.
3. *r* ≥ 0, is the attachment/detachment force between any two existing consecutive particles.
4. *q* ≥ 0, is the attachment/detachment force between any two new consecutive particles.

Each parameter λ_*i*_ and *α*_*i*_ has units of 1/time. A parameter *q* controls the repelling or binding forces between two new neighbors and a parameter *r* between two existing neighbors. In many studies, creating and breaking of bonds between the nearest neighbors has been viewed as opposite chemical reactions [24]. So, it is assumed that $\frac{q}{r}=\text{exp}\left(\frac{E}{K_{B}\;T}\right)$, where *E* denotes the interaction energy, by applying the detailed balance arguments.

The position of the particle along the lattice is denoted by the site covered by the leftmost end of it and this part is referred as the reader. Thus, ‘the reader is at site *i*’ means that the particle is located at site *i* and covers the sites *i*, *i* + 1, …, *i* + *ℓ* − 1. Let *x*_*i*_(*t*) ∈ [0, 1] denote the normalized reader density of the biological particle at site *i* at time *t* and let *y*_*i*_(*t*) ∈ [0, 1] denote its normalized coverage density at site *i* at time *t*, i.e.,

$$\begin{array}{c} y_{i}\left(t\right)=\sum_{j=max\{1,i-\ell +1\}}^{i}x_{j}\left(t\right),\;\;\;i=1,2,\ldots ,n. \end{array}$$ (1)

**Fig 1:**
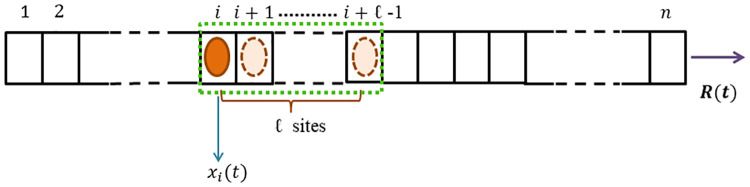
A schematic view of a single particle of size *ℓ* at site *i* covering sites *i*, *i* + 1, …, *i* + *ℓ* − 1 on the lattice of dimension n. The state variable *x*_*i*_(*t*) describes the reader density of particle at site *i* at time *t*. *R*(*t*) denotes the output rate at time *t*.

The term ‘normalized’ here means that each *x*_*i*_(*t*) and each *y*_*i*_(*t*) takes value in the interval [0, 1] for all *t* ≥ 0. The value zero [one] corresponds to completely empty [full]. The schematic explanation of a particle with size *ℓ* on the lattice is shown in Fig 1.

**Fig 2:**
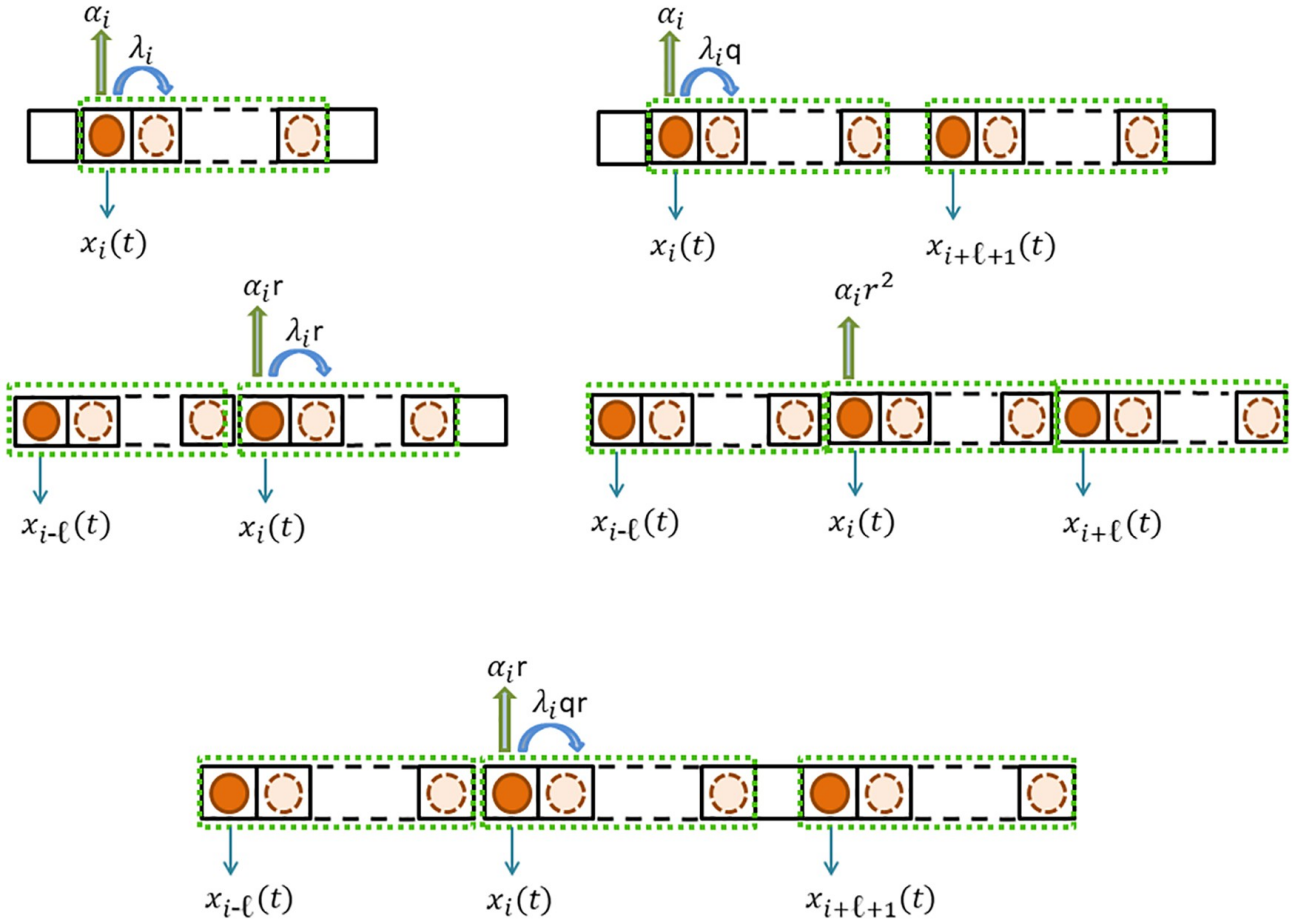
The particle covers *ℓ* sites and the dark red label denotes the reader location. Schematic explanation of the transition flow from site *i* to site *i* + 1 in the EFEIOD: Upper-left: When there are no readers at sites *i* − *ℓ*, *i* + *ℓ* and *i* + *ℓ* + 1, the transition rate is λ_*i*_ and detachment rate is *α*_*i*_. Upper-right: When there is a reader at site *i* + *ℓ* + 1 and site *i* − *ℓ* does not have, the transition rate is λ_*i*_*q* and detachment rate is *α*_*i*_. Middle-left: When there is reader at site *i* − *ℓ* and no readers at sites *i* + *ℓ* and *i* + *ℓ* + 1, the transition rate is λ_*i*_*r* and detachment rate is *α*_*i*_*r*. Middle-right: When there are readers at sites *i* − *ℓ* and *i* + *ℓ*, detachment rate is *α*_*i*_*r*^2^. Lower part: When there are readers at sites *i* − *ℓ* and *i* + *ℓ* + 1, the transition rate is λ_*i*_*qr* and detachment rate is *α*_*i*_*r*.

Eq (1) implies that the total particle coverage at any site *i* is the summation of the reader densities of *ℓ* consecutive sites left to site *i*. The state variables *x*_*i*_(*t*) and *y*_*i*_(*t*) can be interpreted as the probability that site *i* is occupied and covered respectively at time *t*. Hence, *x*_*i*_ and *y*_*i*_ are dimensionless. Fig 2 depicts the possible transition scenarios from site *i* to site *i* + 1.

To state the dynamical equations describing the EFEIOD, we introduce more notation for simplicity. Let

$$\begin{array}{c} z_{i}\left(t\right)≔\{\begin{array}{cc} x_{i}\left(t\right) & i=1,2,\ldots ,n\\ 0 & \text{otherwise}, \end{array} \end{array}$$ (2)

and

$$\begin{array}{c} w_{i}\left(t\right)≔\{\begin{array}{cc} y_{i}\left(t\right) & i=1,2,\ldots ,n\\ 0 & \text{otherwise} \end{array} \end{array}$$ (3)

The dynamics of the EFEIOD is described by *n* nonlinear first order ordinary differential equations:

$$\begin{array}{c} {\dot{x}}_{i}=f_{i-1}\left(x\right)-f_{i}\left(x\right)-g_{i}\left(x\right),\;i=1,2,\ldots ,n. \end{array}$$ (4)

where

$$\begin{array}{c} f_{0}\left(x\right)≔\lambda_{0}\left(1-w_{\ell }\right)\left(1+\left(q-1\right)z_{\ell +1}\right), \end{array}$$ (5)

$$\begin{array}{c} f_{i}\left(x\right)≔\lambda_{i}x_{i}\left(1-w_{i+\ell }\right)\left(1+\left(q-1\right)z_{i+\ell +1}\right)\left(1+\left(r-1\right)z_{i-\ell }\right),\;i=1,2,\ldots ,n, \end{array}$$ (6)

and

$$\begin{array}{c} g_{i}\left(x\right)≔\alpha_{i}x_{i}\left(1+\left(r-1\right)z_{i+\ell }\right)\left(1+\left(r-1\right)z_{i-\ell }\right),\;i=1,2,\ldots ,n. \end{array}$$ (7)

Eq (4) implies that the change in the reader density at site *i* is the inflow *f*_*i*−1_(*x*) from site *i* − 1 to site *i* minus the outflow *f*_*i*_(*x*) to site *i* + 1 minus the outflow *g*_*i*_(*x*) to the cell environment.

Eq (6) can be explained as follows. The term *x*_*i*_ represents that the reader flow from site *i* to site *i* + 1 increases with the reader density at site *i*. The term (1 − *w*_*i*+*ℓ*_) represents a “soft” version of the simple exclusion principle which implies that the flow increases with the ‘vacancy’ level at site *i* + *ℓ* i.e. as the density in any of the *ℓ* consecutive sites increases the reader flow from site *i* to site *i* + 1 gradually decreases. The term (1 + (*q* − 1)*z*_*i*+*ℓ*+1_) represents that the reader flow from site *i* to site *i* + 1 also depends upon the reader density at site *i* + *ℓ* + 1 and increases [decreases] if *q* > 1 [*q* < 1]. The particle at site *i* + *ℓ* + 1 will attract [*q* > 1] or repel [*q* < 1] the particle that move from site *i* to *i* + 1. Similarly, the term (1+ (*r* − 1)*z*_*i*−*ℓ*_) represents that the flow into site *i* + 1 also depends upon the reader density at site *i* − *ℓ*.

The term *g*_*i*_(*x*) in Eq (7) represents the detachment of particles from the site *i* to the cell environment. If *r* > 1 [*r* < 1] then the particles at sites *i* − *ℓ* and *i* + *ℓ* repel [attract] the particle at site *i* and increases [decreases] its detachment from site *i*.

The output rate from site *n* at any time *t* is given by

$$\begin{array}{c} R\left(t\right)=\left(\lambda_{n}+\alpha_{n}\right)x_{n}\left(t\right)\left(1+\left(r-1\right)x_{n-\ell }\right). \end{array}$$ (8)

Note that in the particular case, *r* = 1, *q* = 1, *ℓ* = 1 and *α*_*i*_ = 0, the model gets reduced to the RFM [17]. Clearly, in the case when the length of the biological particle is equal to the lattice length, i.e. *ℓ* = *n*, there are no role of interaction forces in the system. The splitting of interaction energy *E* between the creation and breaking processes is not unique. Like in [15], we also assume that *E* is equally split between the rates *r* and *q*.

$$\begin{array}{c} q=\text{exp}(\frac{E}{2K_{B}T}),\;r=\text{exp}(\frac{-E}{2K_{B}T}) \end{array}$$ (9)

Note that Eq (9) implies *r* = 1/*q* and have a simple physical meaning. If *E* > 0 then there are attractive interactions in the system, i.e. the particle moves faster creating a new pair [*q* > 1] and the process of breaking out of the pair is slowed down [*r* < 1]. Similarly, *E* < 0 implies that there are repulsive interactions in the system. The case *E* = 0 corresponds to the fact that there are no interactions in the system and then we have *q* = *r* = 1.

The next section analyzes the EFEIOD using tools from systems and control theory and in particular contraction theory.

## 3 Main results

Let *C*^*n*^ denote the closed *n*-dimensional unit cube:

$$\begin{array}{c} C^{n}≔\left\{x=\left(x_{1},x_{2},\ldots ,x_{n}\right)\in R^{n}:x_{i}\in \left[0,1\right]\right\}. \end{array}$$ (10)

Note that the state variable *x*_*i*_ at any time *t* represent a reader density in the range [0, 1].

**Example 1** Consider a EFEIOD with dimension *n* = 6, particle size *ℓ* = 2, rates λ_0_ = 0.01, λ_*i*_ = 1, *α*_*i*_ = 0.1 for *i* = 1, 2, …, *n*, *r* = 2 and *q* = 1/2. Consider an initial condition *x*(0) = [1 0.9 0.5 1 1 1]′. It has been observed that at some time *t* we have *x*(*t*) = [1.0033 0.8907 0.4993 1.0744 0.6954 0.9084]′ (all numbers are four digit accurate).

The set *C*^*n*^ is not an invariant set of the EFEIOD as shown in the above example. Therefore, it is relevant to define a state space which is an invariant set of the dynamics. We assume that any initial condition belongs to the state-space defined as:

$$\begin{array}{c} H≔\{x\in R^{n}:x\in C^{n}\;\;\text{and}\;\;Px\in C^{n}\} \end{array}$$ (11)

where *P* is the lower triangular square matrix of size *n* with all entries zero, except for the entries on the main diagonal and (*ℓ* − 1) diagonals below the main diagonal that are ones. Note that the set *H* is a compact and convex set. We have shown in the following subsections that *H* is a relevant state-space for EFEIOD.

Let *int*(*H*) and ∂*H* denote the interior and boundary of *H* respectively. Let *x*(*t*, *a*) denote the solution of Eq (4) at time *t* ≥ 0 for the initial condition *a* ∈ *H*.

### 3.1 Invariance and persistence

The following result shows that *H* is an invariant set for the dynamics of the EFEIOD.

**Proposition 1** *If a ∈ H then the solution of EFEIOD satisfies x(t, a) ∈ H for all t ≥ 0. For any a ∈ ∂H, x(t, a) ∈ int(H) for t > 0*.

This implies that the trajectories that emanate from the boundary of *H* ‘immediately’ enter the interior of *H*. The next proposition is useful because it shows that the solutions of the EFEIOD get ‘immediately’ uniformly separated from the boundary of *H*.

**Proposition 2**. *For any τ > 0, there exists a compact and convex set H_τ_ that is strictly contained in H such that for any a ∈ H, x(t, a) ∈ H_τ_, for all t ≥ τ*.

This means in particular that for any *τ* > 0 there exists $d=d\left(\tau \right)\in \left(0,\frac{1}{2}\right)$ such that,

$$\begin{array}{c} d\leq x_{i}\left(t,a\right),y_{i}\left(t,a\right)\leq 1-d,\;\;\text{for}\;\text{all}\;\;t\geq \tau ,\;\;\text{for}\;\text{all}\;\;i\;\;\text{and}\;\text{all}\;\;a\in H. \end{array}$$ (12)

This property is useful in analyzing the asymptotic properties of the system dynamics.

### 3.2 Contraction

Differential analysis provides a very useful way to study the behavior of certain non-linear dynamical systems. In particular, contraction theory is based on analyzing the time evolution of the distance between the trajectories that emanate from different initial conditions and have its applications to synchronization and reaction-diffusion partial differential equations [24, 25]. However, for our proposed model, we needed a generalized version of contraction theory that has been defined in [26].

Consider a time-varying dynamical system,

$$\begin{array}{c} \dot{x}\left(t\right)=F\left(t,x\left(t\right)\right), \end{array}$$ (13)

with the state *x* evolving on a compact and convex set $\Omega \subseteq R^{n}$. Let *x*(*t*, *t*_0_, *a*) denote the solution of (13) at time *t* ≥ *t*_0_ with *x*(*t*_0_) = *a*.

We say that (13) is contractive on Ω with respect a norm $|.|:R^{n}\to R_{+}$, if there exists *c* > 0 such that

$$\begin{array}{c} |x\left(t_{2},t_{1},a\right)-x\left(t_{2},t_{1},b\right)|\leq \text{exp}\left(-\left(t_{2}-t_{1}\right)c\right)\left|a-b\right|,\;\;\text{for}\;\text{all}\;\;t_{2}\geq t_{1}\geq 0\;\;\text{and}\;\text{all}\;\;a,b\in \Omega . \end{array}$$ (14)

Now, the following generalization of contraction is required to apply contraction theory to the EFEIOD. The system (13) is said to be contractive after a small overshoot and short transient (SOST) [26] with respect a norm $|.|:R^{n}\to R_{+}$, if for each *ϵ* > 0 and each *τ* > 0 there exists *c* = *c*(*τ*, *ϵ*) > 0 such that

$$\begin{array}{c} |x\left(t_{2}+\tau ,t_{1},a\right)-x\left(t_{2}+\tau ,t_{1},b\right)|\leq \left(1+\epsilon \right)\;\text{exp}\left(-\left(t_{2}-t_{1}\right)c\right)\left|a-b\right|, \end{array}$$ (15)

for all *t*_2_ ≥ *t*_1_ ≥ 0 and all *a*, *b* ∈ Ω.

The next result shows that the EFEIOD satisfies this generalization of contraction. Let ${\left|.\right|}_{1}:R^{n}\to R_{+}$ denote the *L*_1_ norm, i.e. for $x\in R^{n}$, |*x*|_1_ = |*x*_1_|+ |*x*_2_|+ …+ |*x*_*n*_|.

**Proposition 3**. *The EFEIOD is SOST with respect to the L_1_ norm, i.e., for each ϵ > 0 and each τ > 0 there exists c = c(τ, ϵ) > 0 such that*

$$\begin{array}{c} {\left|x\left(t+\tau ,a\right)-x\left(t+\tau ,b\right)\right|}_{1}\leq \left(1+\epsilon \right)\;\text{exp}\;\left(-tc\right){\left|a-b\right|}_{1},\;\;\text{for}\;\text{all}\;\;t\geq 0\;\;\text{and}\;\text{all}\;\;a,b\in H. \end{array}$$ (16)

This means that the EFEIOD is contractive after an arbitrarily small time transient *τ* and with an arbitrarily small overshoot (1 + *ϵ*). This implies that any two initial feasible densities in the EFEIOD evolving in time become ‘more similar’ to each other at an exponential rate.

### 3.3 Global asymptotic stability

Since the convex and compact set *H* is an invariant set of the dynamics, it contains atleast one steady-state *e* [27]. By Proposition 1, we have *e* ∈ *int*(*H*). Using Eq (16) with *b* = *e*, yields the following result.

**Theorem 1**. *Assume that q, r > 0. The EFEIOD admits a globally asymptotically stable steady-state density e ∈ int(H), i.e*. lim_*t*→∞_ *x(t, a) = e, for all a ∈ H*.

This means that, regardless of the initial density, all trajectories emanating from different initial conditions converge to the unique steady-state density that depends on the system parameters: transition rates λ_*i*_*s*, detachment rates *α*_*i*_*s*, interactions determined by *q* and *r*, particle size *ℓ* and length of the chain *n*. The next example demonstrates that the assumption *q*, *r* > 0 is necessary.

**Example 2** Consider the EFEIOD with dimension *n* = 3, particle size *ℓ* = 1.

For *q* = *r* = 0 we have:

$$\begin{array}{c} \begin{array}{cc} {\dot{x}}_{1} & =\lambda_{0}\left(1-x_{1}\right)\left(1-x_{2}\right)-\lambda_{1}x_{1}\left(1-x_{2}\right)\left(1-x_{3}\right)-\alpha_{1}x_{1}\left(1-x_{2}\right)\\ {\dot{x}}_{2} & =\lambda_{1}x_{1}\left(1-x_{2}\right)\left(1-x_{3}\right)-\lambda_{2}x_{2}\left(1-x_{1}\right)\left(1-x_{3}\right)-\alpha_{2}x_{2}\left(1-x_{1}\right)\left(1-x_{3}\right)\\ {\dot{x}}_{3} & =\lambda_{2}x_{2}\left(1-x_{1}\right)\left(1-x_{3}\right)-\lambda_{3}x_{3}\left(1-x_{2}\right)-\alpha_{3}x_{3}\left(1-x_{2}\right). \end{array} \end{array}$$ (17)

Also, for *q* = 1 and *r* = 0, we have:

$$\begin{array}{cc} {\dot{x}}_{1} & =\lambda_{0}\left(1-x_{1}\right)-\lambda_{1}x_{1}\left(1-x_{2}\right)-\alpha_{1}x_{1}\left(1-x_{2}\right)\\ {\dot{x}}_{2} & =\lambda_{1}x_{1}\left(1-x_{2}\right)-\lambda_{2}x_{2}\left(1-x_{1}\right)\left(1-x_{3}\right)-\alpha_{2}x_{2}\left(1-x_{1}\right)\left(1-x_{3}\right)\\ {\dot{x}}_{3} & =\lambda_{2}x_{2}\left(1-x_{1}\right)\left(1-x_{3}\right)-\lambda_{3}x_{3}\left(1-x_{2}\right)-\alpha_{3}x_{3}\left(1-x_{2}\right). \end{array}$$ (18)

Eqs (17) and (18) admits a continuum of steady-states, here [1 1 *v*]′ is a steady-state for all *v*. Therefore, the assumption that *q*, *r* > 0 cannot be dropped.

The next example demonstrates the global asymptotic property, i.e. trajectories starting from different initial conditions in *H* asymptotically converge to a unique density profile along the lattice.

**Fig 3:**
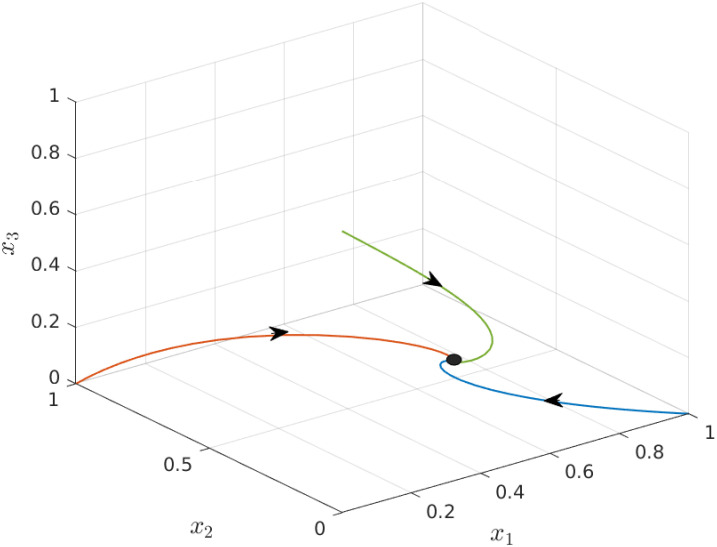
Trajectories of EFEIOD for three initial conditions given in Example 3 as a function of time. The equilibrium point is marked by a ellipse.

**Example 3** Consider the EFEIOD with dimension *n* = 3, particle size *ℓ* = 2, rates λ_*i*_ = 1, *α*_*i*_ = 0.01, *q* = 1 and *r* = 1. Fig 3 depicts trajectories for three different initial conditions [1, 0, 0], [0, 1, 0] and [0, 0, 1] in *H*. It can be seen that the three solutions converge to the same equilibrium point *e* = [0.4959 0.2483 0.2459]′.

The next subsection analyze how the various parameters in the proposed model affects the steady-state output rate.

### 3.4 Analysis of the steady-state

At steady state, for *x* = *e* the left-hand side of all the equations in (4) is zero, so

$$\begin{array}{c} f_{i-1}\left(e\right)=f_{i}\left(e\right)+g_{i}\left(e\right),\;i=1,2,\ldots ,n. \end{array}$$ (19)

It follows from Eq (19) that if we multiply parameters λ_*i*_*s* and *α*_*i*_*s* by a scalar constant *c* > 0 then *e* will not change, i.e. *e*(*cp*) = *e*(*p*) where *p* = [λ_0_, λ_1_, …, λ_*n*_, *α*_1_, *α*_2_, …, *α*_*n*_]. Also, *R*(*cp*) = *cR*(*p*), i.e. the output rate is homogeneous of order one w.r.t. the parameters λ_*i*_*s* and *α*_*i*_*s*. By Eq (19), we have:

$$\begin{array}{c} R=f_{n}\left(e\right)+g_{n}\left(e\right)=f_{i}\left(e\right)-\sum_{k=i+1}^{n-1}g_{k}\left(e\right),\;\;i=0,1,\ldots ,n-1. \end{array}$$ (20)

However, solving Eq (20), in general, is non-trivial.

The next result shows that the derivatives of the equilibrium point coordinates with respect to the rates exists and are well defined. Let the mapping from the parameters to the unique equilibrium point be denoted by *η*, i.e., *η*_*i*_(*γ*) = *e*_*i*_, all *i* = 1, 2 …, *n* and *γ* = [λ_0_, λ_1_, …, λ_*n*_, *α*_1_, *α*_2_, …, *α*_*n*_, *r*, *q*]′.

**Proposition 4** *The derivative (∂/∂γ_j_)η_i_(γ) exists for all i, j*.

The above result allows to calculate the derivatives of the steady-state density if some of the parameters in the system are changed. This is useful to study the sensitivity of the steady-state w.r.t. small changes in the rates.

### 3.5 Effect of interactions

We demonstrate with several simulations the non-trivial effect of interactions on the steady-state of the EFEIOD.

The example below demonstrates that in the presence of strong attractive interactions, detachment of particles could be useful for increasing the flow of particles along the lattice.

**Fig 4:**
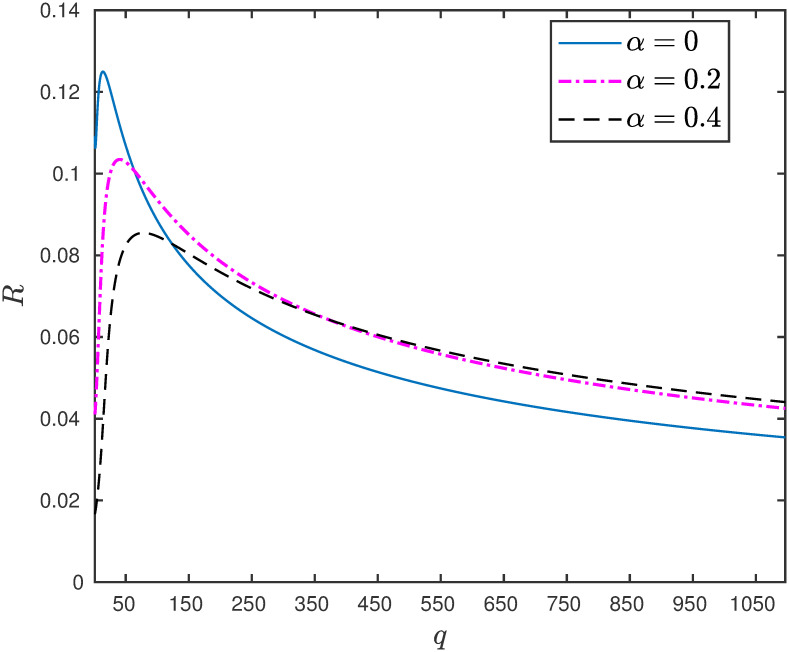
The steady-state output rate *R* as a function of *q* for a EFEIOD with *n* = 9, *ℓ* = 3, λ_0_ = 1, λ_*i*_ = 1, *α*_*i*_ = *α*, for all *i*.

**Example 4** Consider the EFEIOD with dimension *n* = 9, particle size *ℓ* = 3, rates λ_0_ = 1, λ_*i*_ = 1 and *α*_*i*_ = *α*. Fig 4 depicts that increasing the detachment rate increases the steady-state output for the higher values of *q*.

The above example suggests that for larger values of attractive interactions there could be a regulatory mechanism to increase the flow of particles in the system by allowing the particles to detach from the sites. The next example shows the positive role of increasing the detachment rate in the presence of a bottleneck rate at a site.

**Fig 5:**
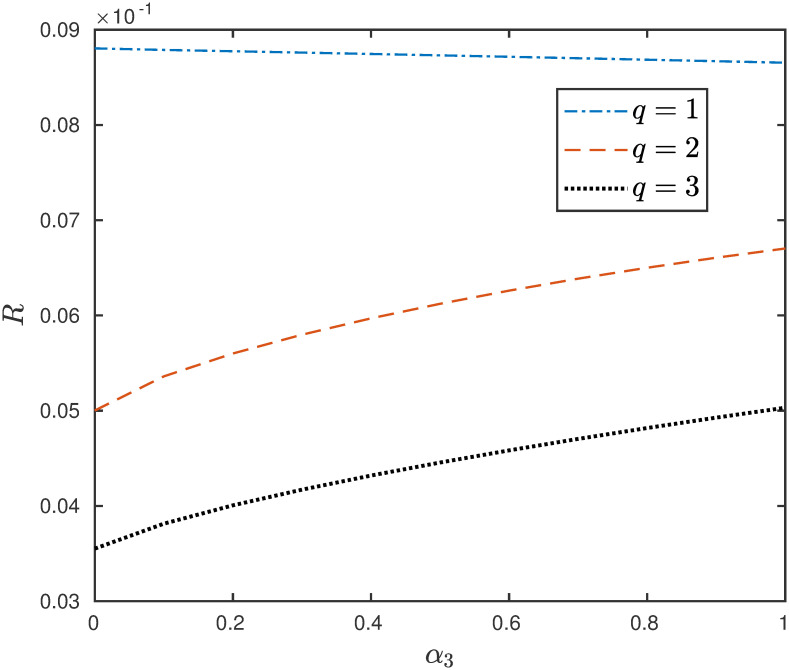
The steady-state output rate *R* as a function of *α*_3_ for a EFEIOD with *n* = 9, *ℓ* = 2, λ_0_ = 1, λ_*i*_ = 1 for all *i* except λ_5_ = 0.01, *α*_*i*_ = 0 and *r* = 1/*q*.

**Example 5** Consider the EFEIOD with dimension *n* = 9, particle size *ℓ* = 2, rates λ_0_ = 1, λ_*i*_ = 1 for all *i* except λ_5_ = 0.01, *α*_*i*_ = 0. Note that λ_5_ is the bottleneck rate. We vary the parameter *α*_3_, i.e. detachment rate of particles at site 3. It can be seen in Fig 5 that for *q* > 1 i.e. *r* < 1, increasing *α*_3_ increases the steady-state output rate. However, for *q* = 1 i.e. *r* = 1, increasing *α*_3_ decreases the steady-state output rate.

This can be explained as follows: for *r* < 1, a particle at site 5 will tend not to hop forward as there is strong attraction from particle at site 3. Therefore, allowing particles to detach from site 3, leads to an easy flow of particles from site 5 and this increases the flow. This is important to study as the interactions from the neighboring particles at the bottleneck rate further deteriorate the flow of particles along the lattice. In the case of no interactions i.e. *q* = 1 [*r* = 1], it can be seen that increasing the detachment rate leads to decrease in the steady-state flow which is always true as we theoretically analyze this special case in the next section.

The example above demonstrates that in the case of interactions, locally controlled detachment can avoid bottlenecks and can lead to faster movement of particles, and hence increasing the flow and alleviating the “traffic jams” [28]. One may perhaps think that increasing the particle size leads to a decrease in the steady-state output rate, but steady-state densities follow complicated behavior in the presence of interactions. It have been shown that when *q* = *r* = 1 and *α*_*i*_s = 0, steady-state output rate for *ℓ* > 1 is always less than steady-state output rate for the RFM [29]. But in the presence of interactions, increasing length does not always decrease the output rate as shown in the example below.

**Fig 6:**
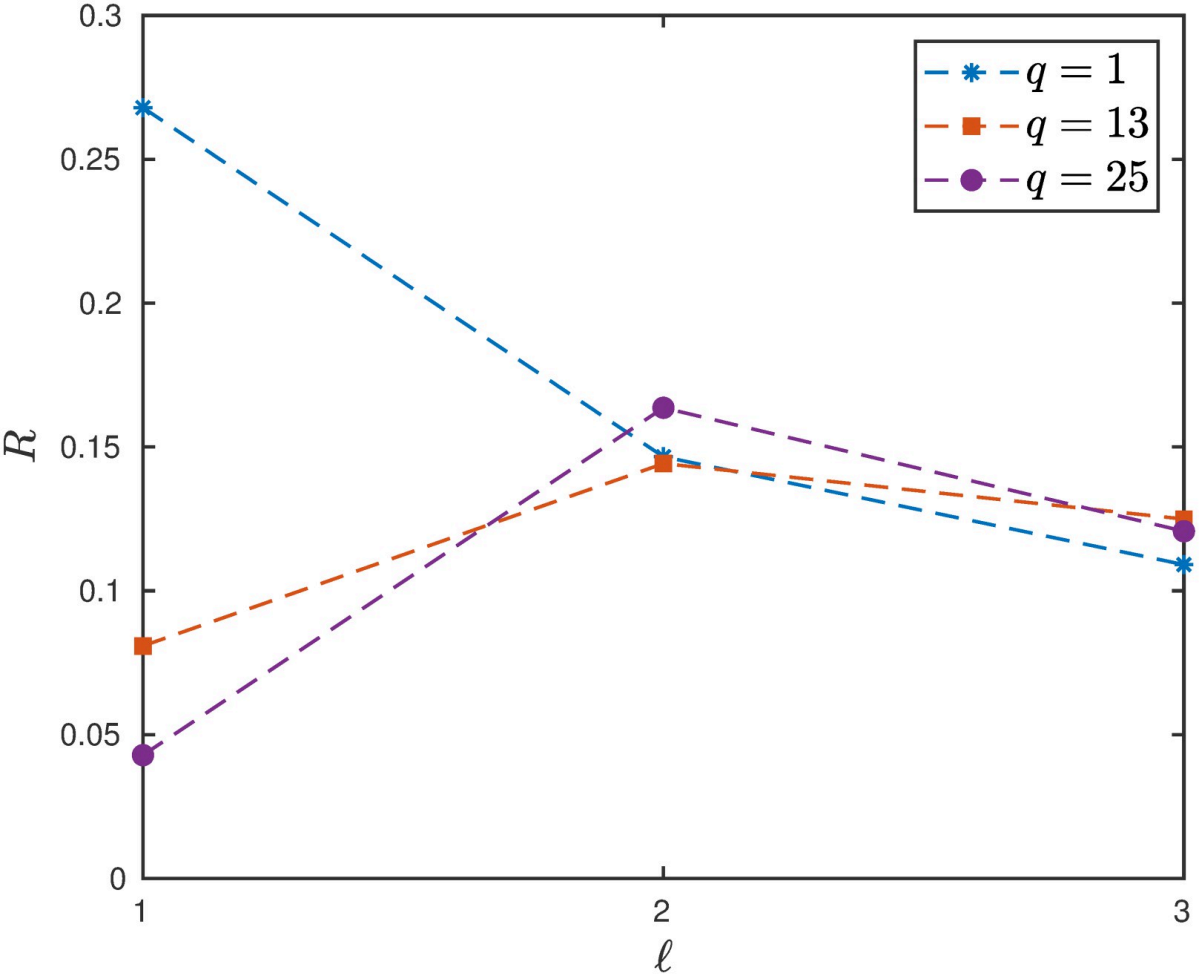
The steady-state output rate *R* as a function of *ℓ* ∈ {1, 2, 3} for a EFEIOD with *n* = 9, λ_0_ = 1, λ_*i*_ = 1, *α*_*i*_ = 0, for all *i* and *r* = 1/*q*.

**Example 6** Consider the EFEIOD with dimension *n* = 9, rates λ_0_ = 1, λ_*i*_ = 1 for all *i*, *α*_*i*_ = 0. We vary the particle size *ℓ*. It can be seen in Fig 6 that for *q* = 13; for *ℓ* = 1, *R* = 0.0808, whereas for *ℓ* = 2, *R* = 0.1442 and for *ℓ* = 3, *R* = 0.1249.

Furthermore, in the thermodynamical limit, i.e. number of sites goes to ∞, the homogeneous case of TASEPEO with strong repulsions and particle size *ℓ*, and with entry and exit rates equal to one is in the maximal current phase, where the steady-state mean reader density $1/(\ell +1+\sqrt{\ell +1})$ and the steady-state output rate is $1/({\left(1+\sqrt{\ell +1}\right)}^{2})$ [14]. This implies that as *ℓ* goes to ∞, the steady-state output and mean reader density go to zero. The next example shows that this is consistent with the results of our model. We define the steady-state mean reader density by $\rho =\left(1/n\right)\sum_{i=1}^{n}e_{i}$.

**Fig 7:**
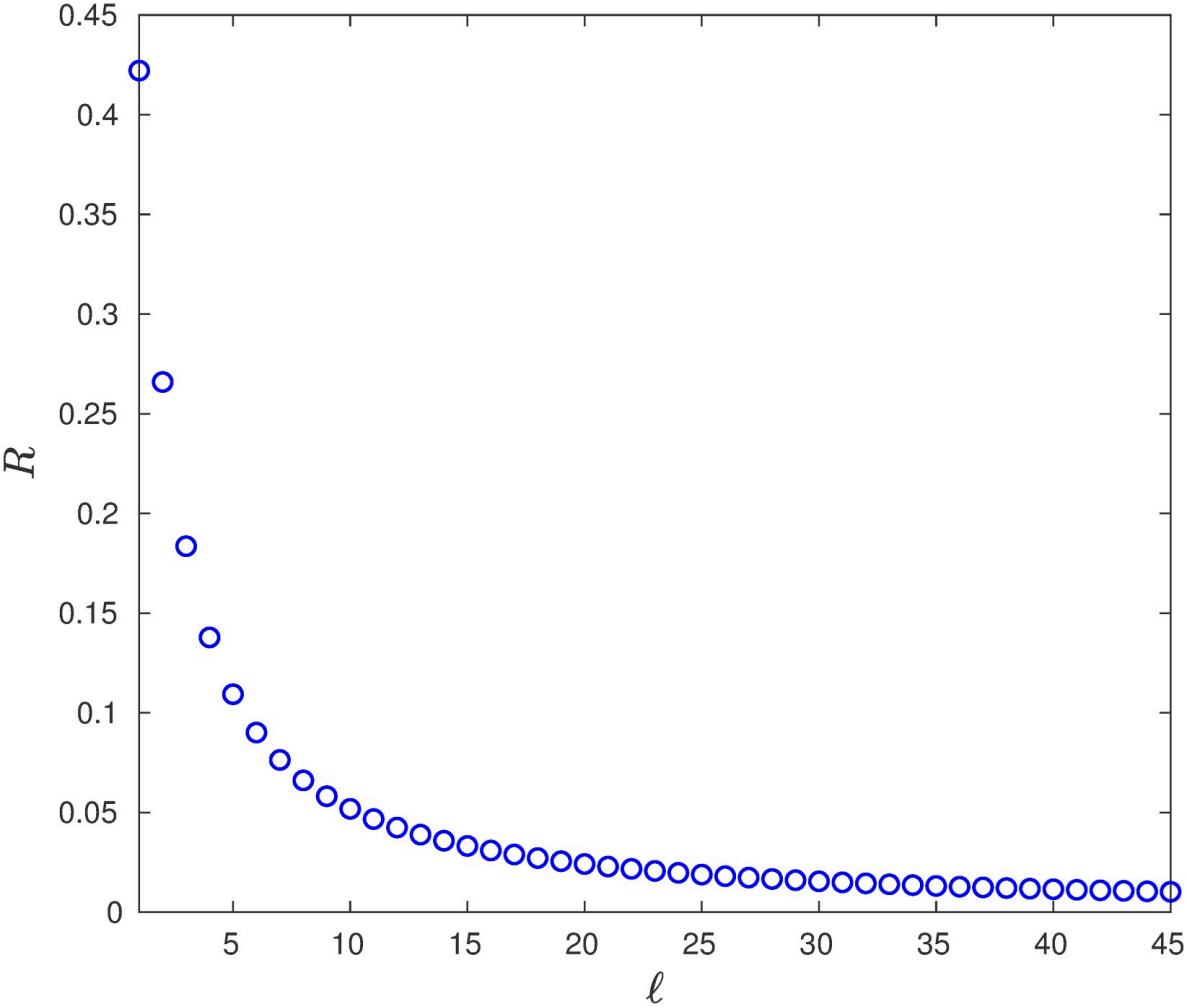
The steady-state output rate *R* as a function of *ℓ* ∈ {1, 2, …, 45} for a EFEIOD with *n* = 100, λ_0_ = 1, λ_*i*_ = 1, *α*_*i*_ = 0, for all *i*, *q* = 0.01 and *r* = 1/*q*.

**Fig 8:**
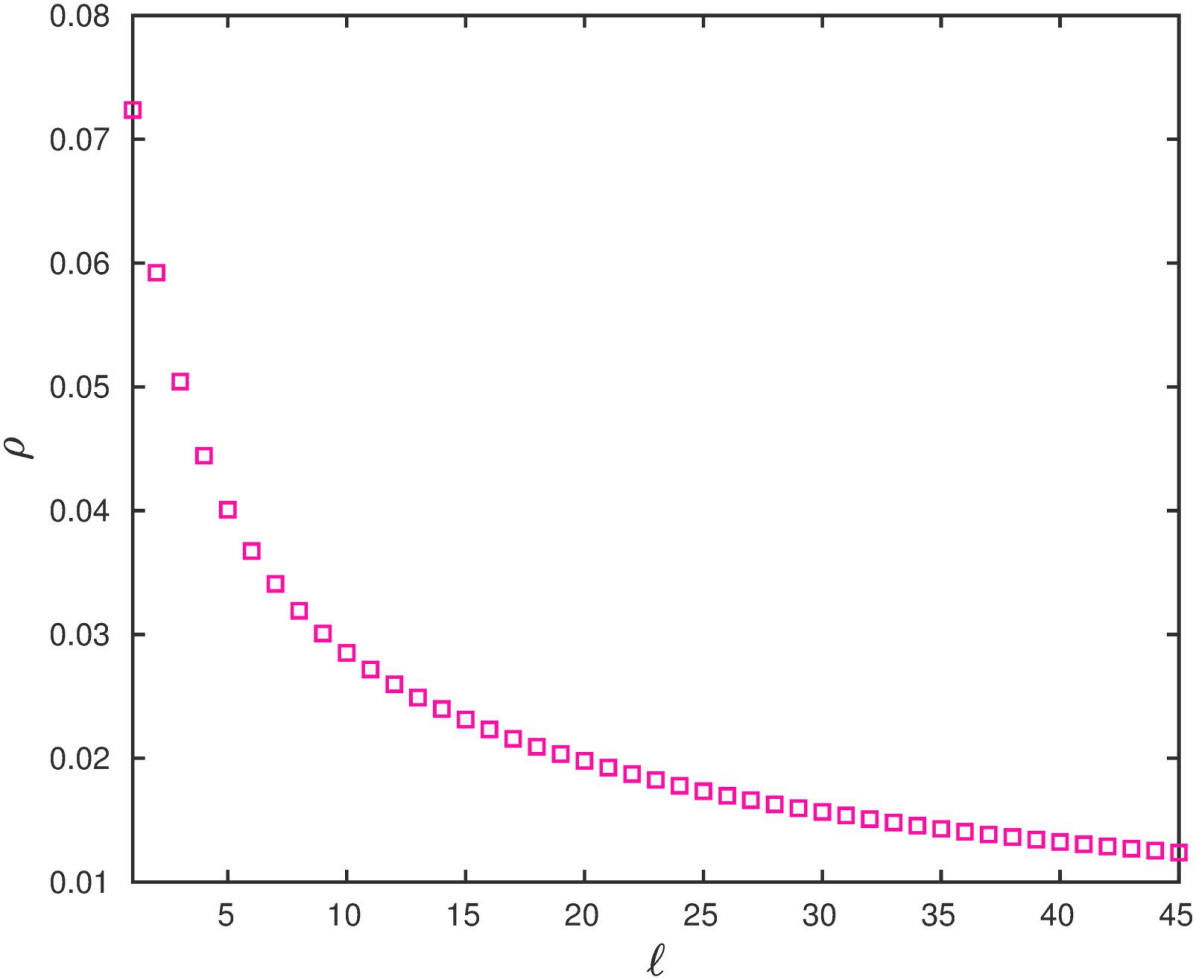
The steady-state mean reader density *ρ* as a function of *ℓ* ∈ {1, 2, …, 45} for a EFEIOD with *n* = 100, λ_0_ = 1, λ_*i*_ = 1, *α*_*i*_ = 0, for all *i*, *q* = 0.01 and *r* = 1/*q*.

**Example 7** Consider EFEIOD with dimension *n* = 100, rates λ_0_ = 1, λ_*i*_ = 1 for all *i*, *α*_*i*_ = 0, *q* = 0.01, *r* = 100 and particle size *ℓ*. Fig 7 depicts that output rate *R* decrease with *ℓ*. Also, the steady-state mean reader density *ρ* decrease with *ℓ* as seen in Fig 8.

The next example shows that in the presence of interactions, increase in an initiation rate does not always lead to increase in the output rate. However, in the case of no interactions i.e. *q* = 1 [*r* = 1], increase in an initiation rate due to feedbacks or due to an increase in number of ‘free’ biological particles leads to an increase in the steady-state output rate as we theoretically analyze this special case in the next section.

**Fig 9:**
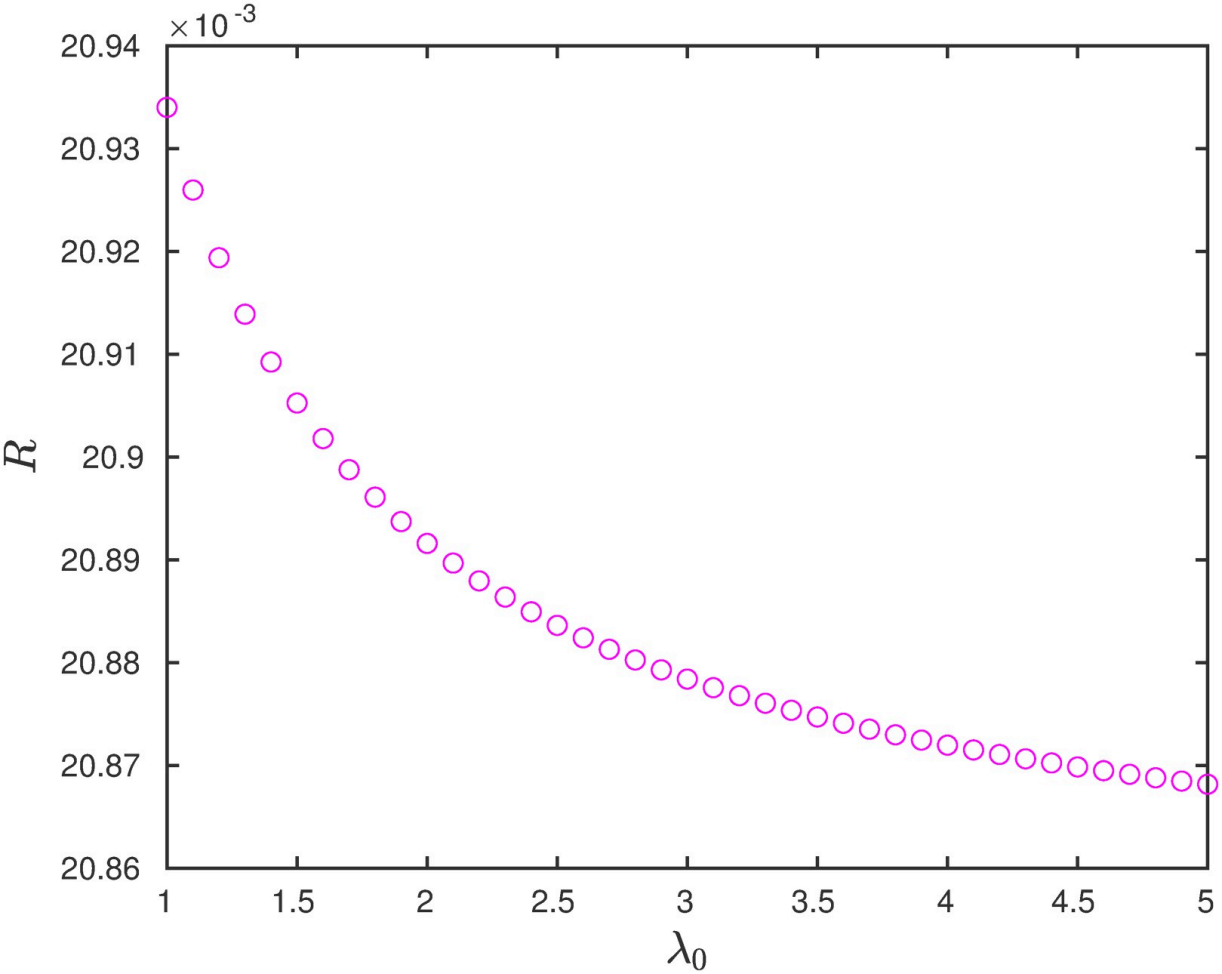
The steady-state output rate *R* as a function of λ_0_ for a EFEIOD with *n* = 6, *ℓ* = 2, λ_0_ = 1, λ_*i*_ = 1, except λ_4_ = 0.1, *α*_*i*_ = 0, for all *i*, *q* = 7 and *r* = 1/7.

**Fig 10:**
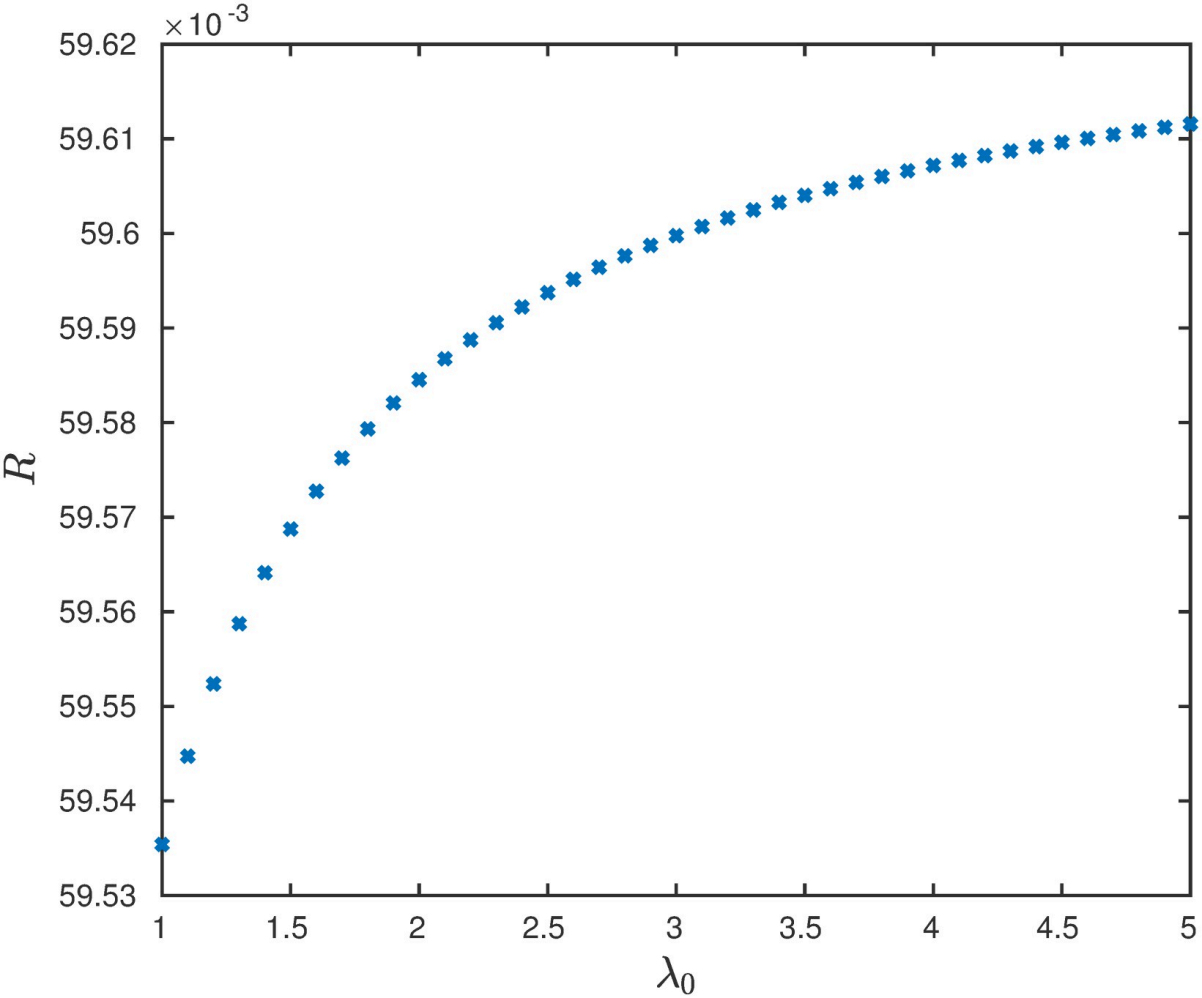
The steady-state output rate *R* as a function of λ_0_ for a EFEIOD with *n* = 6, *ℓ* = 2, λ_0_ = 1, λ_*i*_ = 1, except λ_4_ = 0.1, *α*_*i*_ = 0, for all *i*, *q* = 1 and *r* = 1.

**Example 8** Consider the EFEIOD with dimension *n* = 6, *ℓ* = 2, rates λ_*i*_ = 1 for all *i* except λ_4_ = 0.1 and *α*_*i*_ = 0. We vary the initiation rate λ_0_. It can be seen in Fig 9 that steady-state output rate decreases with increase in λ_0_. Fig 10 depicts that the steady-state output rate increases with increase in λ_0_.

Now, we analyze the effect of increasing the length of particle in the case *q* → ∞.

**Fig 11:**
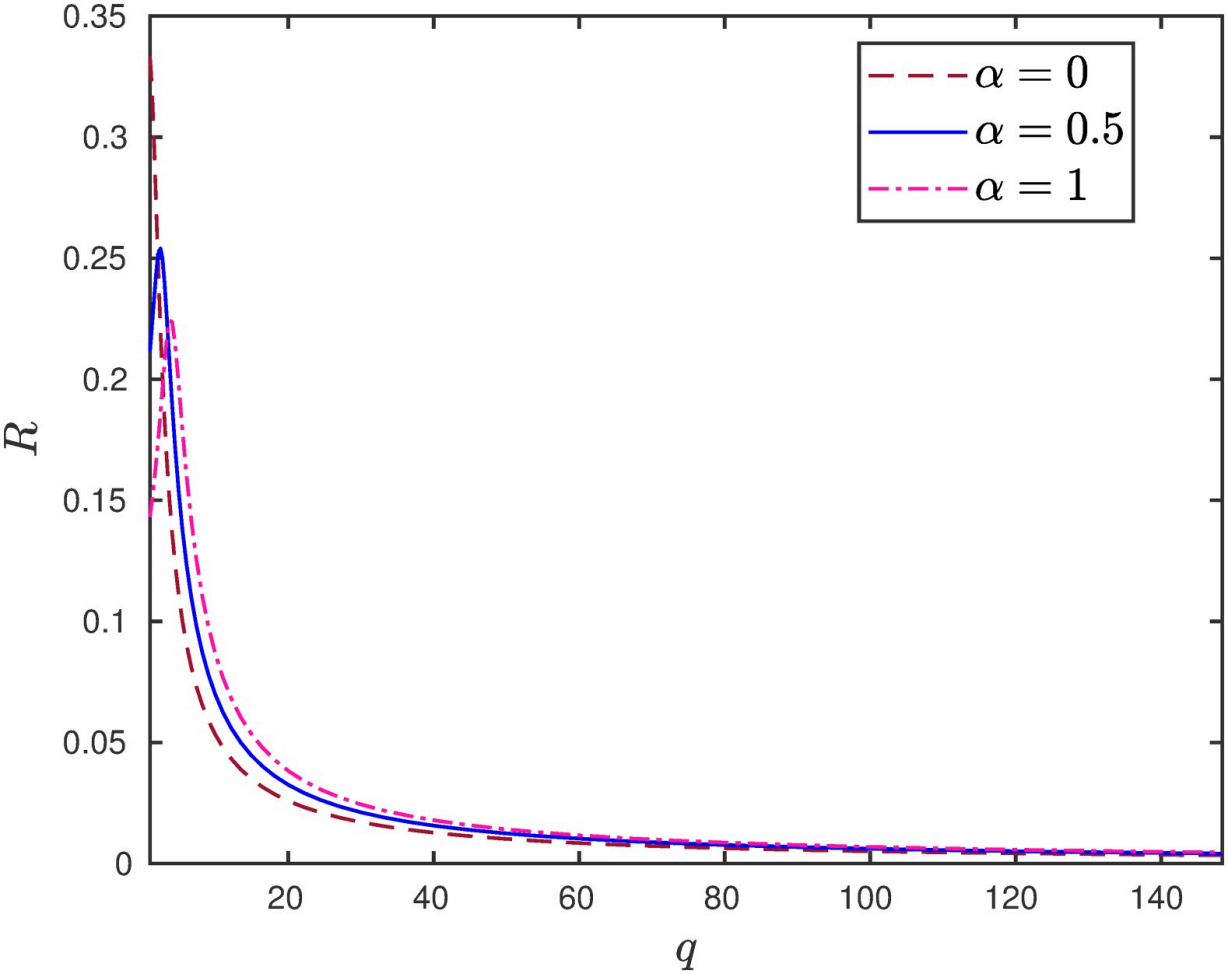
The steady-state output rate *R* as a function of *E* for a EFEIOD with *n* = 3, *ℓ* = 1, λ_0_ = 1, λ_*i*_ = 1, *α*_*i*_ = *α*, for all *i* and *r* = 1/*q*.

**Fig 12:**
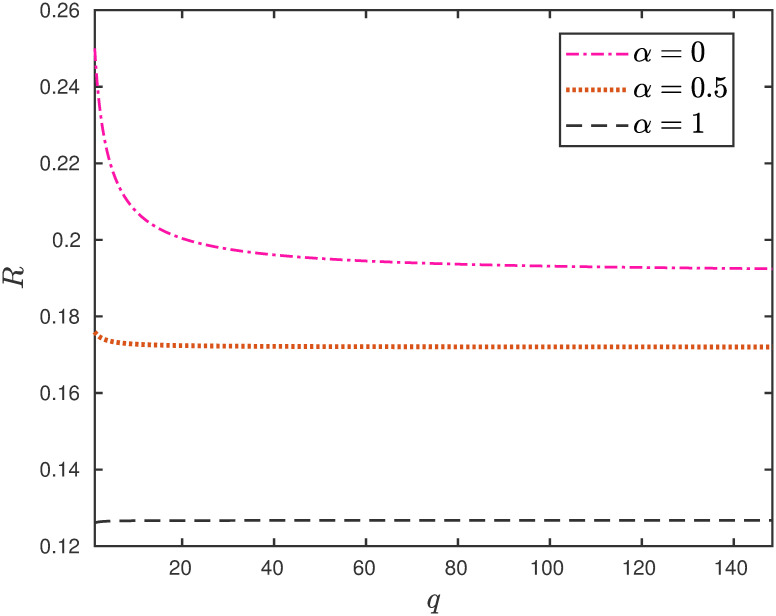
The steady-state output rate *R* as a function of *E* for a EFEIOD with *n* = 3, *ℓ* = 2, λ_0_ = 1, λ_*i*_ = 1, *α*_*i*_ = *α*, for all *i* and *r* = 1/*q*.

**Example 9** Consider the EFEIOD with dimension *n* = 3, λ_0_ = 1, λ_*i*_ = 1, *α*_*i*_ = *α*, for all *i*. Fig 11 depicts that when *q* → ∞, the steady-state output rate decrease to zero. Fig 12 depicts that when *q* → ∞, the steady-state output rate saturates to a non-zero constant value depending on the value of *α*, i.e. *R* = 0.1910 for *α* = 0, *R* = 0.1720 for *α* = 0.5 and *R* = 0.1267 for *α* = 1.

A high value of *q* corresponds to a strong attachment between existing neighbors (small *r*) and a high tendency for creating new neighbors (large *q*), resulting in traffic jams and leading to a sharp decrease in the output rate. Therefore, the length of the particle has an interesting role to play in order to maintain a non-zero constant steady-state output rate in the case of weak repulsions.

### 3.6 Entrainment

There are many biological processes that are periodic [30], for example in translation-elongation mechanism; tRNA molecules [31], ATP levels [32], ribosome drop-off rate [33], translation initiation and elongation factors [34], oscillations in mRNA levels [35] and more may vary in a periodic manner and this results into the periodicity of the rates in the system. For the proper functioning of our body, certain biological systems must be in sync with the periodic changes induced due to the continuously changing environment [36, 37]. Entrainment also plays an important part in designing extracellular biomedical systems [38]. An important question is: will the state variables of EFEIOD preserve the property of entrainment w.r.t. the parameters λ_*i*_s, *α*_*i*_s, *q* and *r*?

Assume that the λ_*i*_s, *α*_*i*_s, *q* and *r* are non-negative, uniformly bounded time-varying continuous functions satisfying:

- There exists a (minimal) *T* > 0 such that every λ_*i*_s, *α*_*i*_s, *q* and *r* is a *T*-periodic function.
- There exists 0 < *δ*_1_ < *δ*_2_ such that λ_*i*_(*t*) ∈ [*δ*_1_, *δ*_2_], for all *i* = 0, 1, …, *n* and all *t* ≥ 0.

This model have been referred as periodic EFEIOD (PEFEIOD). The next result follows from the fact that EFEIOD is SOST on *H* and the known results on entrainment [39].

**Theorem 2** *The PEFEIOD admits a unique function* ϕ:R→int(H) , *that is T-periodic and for any initial condition a ∈ H, the trajectory x(t, a) converges asymptotically to φ*.

The above theorem implies that the state variables entrain to the periodic excitations in the parameters. The next example illustrates the behavior of PEFEIOD.

**Fig 13:**
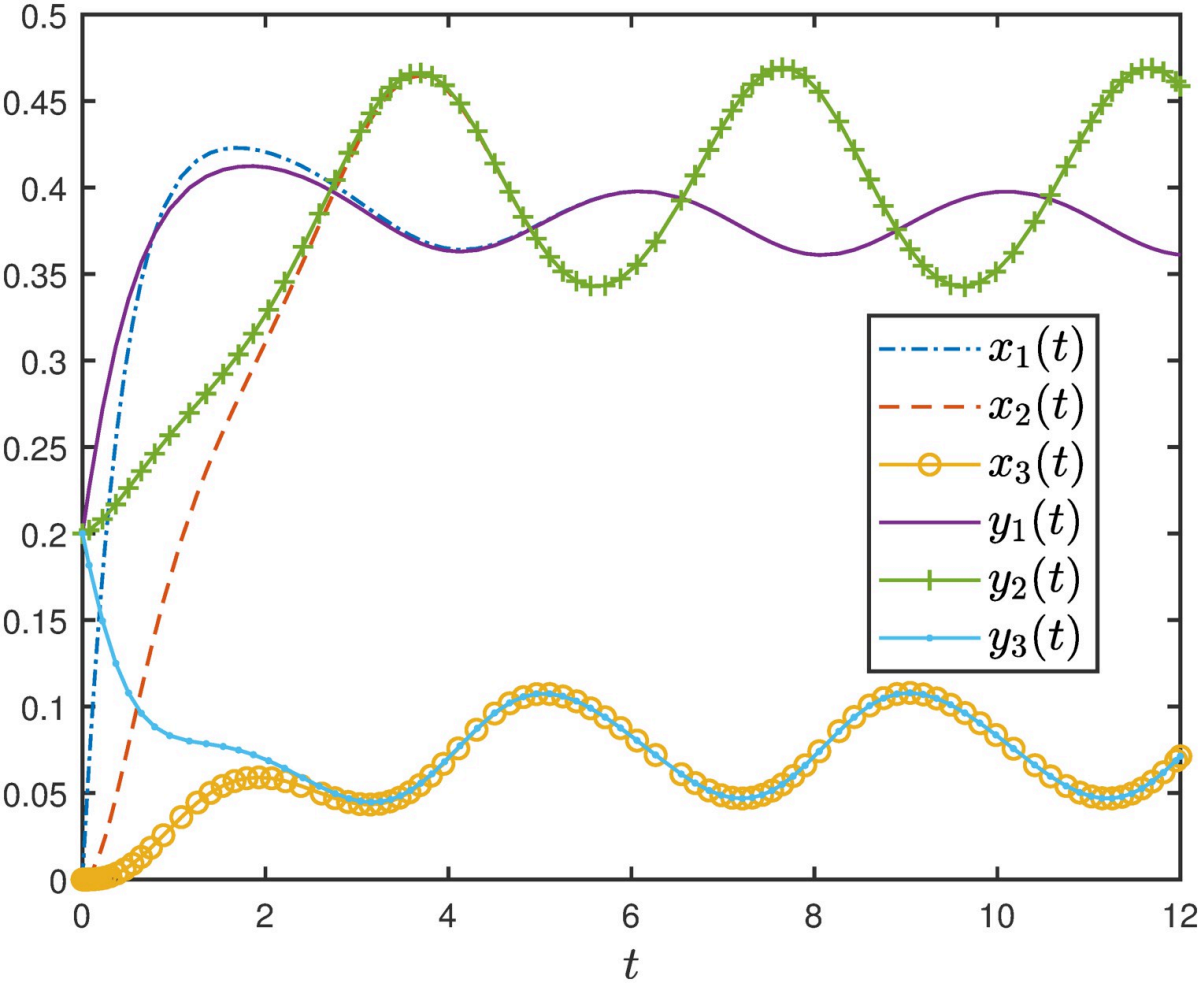
Trajectories of PEFEIOD in Example 10 as a function of time (*t*). Here, *x*_*i*_(*t*) and *y*_*i*_(*t*) are the trajectories of PEFEIOD corresponding to initial conditions [0, 0, 0] and [0.2, 0.2, 0.2] respectively.

**Example 10** Consider a PEFEIOD with dimension *n* = 3, ribosome size *ℓ* = 2, λ_0_ = 1 λ_*i*_ = 1, except for λ_2_(*t*) = 0.5 + 0.25 *sin*(*πt*/2) *α*_*i*_ = 0.01, *r* = 5 and *q* = 1/5. Note, that there is single time-varying periodic rate in the network and all these rates are periodic with a common minimal period *T* = 4. We have taken two different initial conditions [0, 0, 0] and [0.2, 0.2, 0.2] in *H*. It can be seen from the Fig 13 that all the trajectories converge to the periodic solution with period *T* = 4.

In general, to describe the effect of parameters on the system dynamics by a theoretical framework is cumbersome, as analyzing the set of non-linear equations that define the steady-state is not trivial. However, for a special case *q* = *r* = 1, the steady-state output rate sensitivity to variations in the parameters of the system can be answered rigorously. Moreover, the proposed general model was representative of the biology of molecular motors whereas this special case is important in the context of studying the ribosome flow and provides a tool in developing a better understanding and analyzing the factors that can affect this dynamical process of translation.

## 4 Ribosome flow model with extended objects and ribosome drop-off

The synthesis of protein as directed by the mRNA template consisting of codons is carried out by ribosome and the process is referred to as translation [40]. The process broadly takes place in three steps: initiation where ribosomal complex assembles at the start codon of an mRNA chain; elongation where it moves along the mRNA in a forward series of steps forming a polypeptide chain of amino acids, and termination where it releases the chain that folds into functional protein and unbinds from the mRNA. Translation is a fundamental cellular process that occurs in all living beings at all times [41] and is known to consume most of the cell’s energy [42]. Therefore, it is crucial to understand its dynamical aspects through mathematical modeling [43–46].

It is known from previous studies that the footprint of the ribosome on the mRNA is 10 to 20 codons [47–49]. Many ribosomes can simultaneously move on the same mRNA template, blocking the movement of other ribosomes behind it [50], resulting in traffic-like movement on the template, and these “traffic jams” are more severe in genes that are lowly expressed [51]. The ribosomes that initiate translation of mRNA sequence may not successfully complete it and hence fails to produce a full-length protein product [52, 53]. Hence, there are translational errors that can disrupt cellular fitness and can cause diseases [54]. Such errors can have multiple causes like ribosomal traffic jams, reading frameshifts [55], non-availability of tRNAs [56], misreading of codon, premature stop codons [57–59], etc. These errors often result in ribosome dissociating from the mRNA before reaching the stop codon called ribosome drop-off event, resulting in incomplete or incorrect peptides that are mostly non-functional, possibly toxic to the cell. The translational error due to premature translation termination seems to represent more than two-thirds of the overall errors and thus have a strong impact on protein formation [60, 61]. Therefore, modeling mRNA translation with ribosome drop-off is important in analyzing the effect on the translation phenomena as it leads to a reduction in the rate of protein production.

To gain insights into these dynamical aspects of translation, we consider a special case of our model when *q* = *r* = 1 and we refer to this case as the *ribosome flow model of extended objects with drop-off effect* (RFMEOD). In this model, mRNA is treated as a one-dimensional lattice of length *n*, where *n* denotes the number of sites (codons) and every ribosome covers 1 ≤ *ℓ* ≤ *n* sites. The site 1 and *n* represents the start and stop codon respectively. The position of the ribosome along the mRNA is denoted by the site covered by the leftmost end of it. At any time *t*, if the leftmost edge of the ribosome is at site *i*, it means the reader is located at site *i* and the ribosome is translating site *i* and sites *i*, …, *i* + *ℓ* − 1 are covered by this ribosome. Ribosomes move unidirectionally from left to right by only one site on the template and no two ribosomes can occupy or cover the same site simultaneously.

The dynamics of RFMEOD is given by:

$$\begin{array}{c} \begin{array}{cc} {\dot{x}}_{1} & =\lambda_{0}\left(1-y_{\ell }\right)-\lambda_{1}x_{1}\left(1-y_{\ell +1}\right)-\alpha_{1}x_{1},\\ {\dot{x}}_{2} & =\lambda_{1}x_{1}\left(1-y_{\ell +1}\right)-\lambda_{2}x_{2}\left(1-y_{\ell +2}\right)-\alpha_{2}x_{2},\\ \vdots \\ {\dot{x}}_{n-\ell +1} & =\lambda_{n-\ell }x_{n-\ell }\left(1-y_{n}\right)-\lambda_{n-\ell +1}x_{n-\ell +1}-\alpha_{n-\ell +1}x_{n-\ell +1},\\ {\dot{x}}_{n-\ell +2} & =\lambda_{n-\ell +1}x_{n-\ell +1}-\lambda_{n-\ell +2}x_{n-\ell +2}-\alpha_{n-\ell +2}x_{n-\ell +2},\\ \vdots \\ {\dot{x}}_{n} & =\lambda_{n-1}x_{n-1}-\lambda_{n}x_{n}-\alpha_{n}x_{n}. \end{array} \end{array}$$ (21)

The term λ_*i*−1_*x*_*i*−1_(1 − *y*_*i*+*ℓ*−1_) represents the reader flow from site *i* − 1 to site *i*. The flow increases with density level of readers at site *i* − 1 and decreases with coverage density *y*_*i*+*ℓ*−1_ = *x*_*i*_ + *x*_*i*+1_ + … + *x*_*i*+*ℓ*−1_. The term *α*_*i*_*x*_*i*_ represents the detachment of particles from the site *i* to the cell environment. Also, the equations describing the last *n* − *ℓ* + 2 equations are linear, as a ribosome reading the last *ℓ* codons is the last particle and hence it move without any hindrance towards the last(stop) codon.

The output rate from site *n* at any time *t*, which is the protein production rate is given by

$$\begin{array}{c} R\left(t\right)=\left(\lambda_{n}+\alpha_{n}\right)x_{n}\left(t\right). \end{array}$$ (22)

### 4.1 Analysis of the steady-state

The RFMEOD Eq (21) can be written as

$$\begin{array}{c} {\dot{x}}_{i}=f_{i-1}\left(x\right)-f_{i}\left(x\right)-g_{i}\left(x_{i}\right),\;i=1,2,\ldots ,n, \end{array}$$ (23)

where

$$\begin{array}{cc} f_{0}\left(x\right) & ≔\lambda_{0}\left(1-y_{\ell }\right),\\ f_{i}\left(x\right) & ≔\lambda_{i}x_{i}\left(1-y_{i+\ell }\right),\;i=1,\ldots ,n-1,\\ f_{n}\left(x\right) & ≔\lambda_{n}x_{n},\\ g_{i}\left(x_{i}\right) & ≔\alpha_{i}x_{i}. \end{array}$$ (24)

Also, *y*_*i*_ = 0 for all *i* ≥ *n* + 1. At steady state, the left-hand side of the Eq (23) is zero, so

$$\begin{array}{c} f_{i-1}\left(e\right)=f_{i}\left(e\right)+g_{i}\left(e_{i}\right),\;\;i=1,2,\ldots ,n. \end{array}$$ (25)

Let *R* = (λ_*n*_ + *α*_*n*_)*e*_*n*_ denote the steady-state output rate. From Eq (25), we get

$$\begin{array}{c} R=f_{n}\left(e\right)+g_{n}\left(e_{n}\right)=f_{i}\left(e\right)-\sum_{k=i+1}^{n-1}g_{k}\left(e_{k}\right),\;\;i=0,1,\ldots n-1. \end{array}$$ (26)

This yields the following set of *n* + 1 equations in the *n* + 1 unknowns: *e*_1_, …, *e*_*n*_, *R*:

$$\begin{array}{cc} e_{n} & ≔\frac{R}{\lambda_{n}},\\ e_{i} & ≔\frac{R+\sum_{k=i+1}^{n-1}g_{k}\left(e_{k}\right)}{\lambda_{i}\left(1-y_{i+\ell }\right)},\;i=n-1,\ldots ,1,\\ \text{and}\;\text{also}\\ y_{\ell } & ≔\frac{\lambda_{0}-R-\sum_{k=1}^{n-1}g_{k}\left(e_{k}\right)}{\lambda_{0}}. \end{array}$$ (27)

Solving Eq (27) is in general non-trivial. Nevertheless, it can be solved in closed form in some special cases. Note that when *α*_*i*_ = 0 for all *i*, RFMEOD gets reduced to RFMEO [29].

**Example 11** Consider a RFMEOD with dimension *n* and with particle size *ℓ* = *n*. Consider homogeneous rates; λ_*i*_ = λ, for *i* = 0, 1, …, *n* and *α*_*i*_ = *α*, *i* = 1, 2, …, *n*. We have,

$$\begin{array}{c} R=\frac{\lambda }{1+\frac{\lambda }{\lambda +\alpha }+\left(\frac{\lambda +\alpha }{\lambda }\right)\left(\sum_{i=1}^{n-1}\sum_{j=0}^{n-i-1}(\begin{array}{c} n-i-1\\ j \end{array}){\left(\frac{\alpha }{\lambda }\right)}^{j}\right)}, \end{array}$$ (28)

and

$$\begin{array}{c} e_{i}=\frac{\sum_{j=0}^{n-i-1}(\begin{array}{c} n-i-1\\ j \end{array}){\left(\frac{\alpha }{\lambda }\right)}^{j}}{1+\frac{\lambda }{\lambda +\alpha }+\left(\frac{\lambda +\alpha }{\lambda }\right)\left(\sum_{i=1}^{n-1}\sum_{j=0}^{n-i-1}(\begin{array}{c} n-i-1\\ j \end{array}){\left(\frac{\alpha }{\lambda }\right)}^{j}\right)}. \end{array}$$ (29)

In case of totally homogeneous rates λ = *α* we have,

$$\begin{array}{c} R=\frac{2\;\lambda }{2^{n+1}-1},\;\;\text{and}\;\;e_{i}=\frac{2^{n-i}}{2^{n+1}-1}. \end{array}$$ (30)

Eq (26) can be used to prove various theoretical results. The next result shows that increasing any of the *α*_*i*_*s*, *i* ≠ *n*, decreases R. In other words increasing any of the internal detachment rate decreases the steady-state protein production rate.

**Proposition 5**. *Consider a RFMEOD with dimension n and with particle size ℓ. Then (∂/∂α_i_)R < 0, for all* *i* = 1, 2, …, *n* − 1.

The next result shows that increasing any of the λ_*i*_*s* increases R. In particular, increasing the initiation rate always leads to an increase in protein synthesis rate. This result is consistent with a proposed canonical model of eukaryotic translation exhibiting a relation between initiation rate and protein expression [62].

**Proposition 6** *Consider a RFMEOD with dimension n and with particle size ℓ. Then (∂/∂λ_i_)R > 0, for* *i* = 0, 1, 2, …, *n*.

Consider the case where all λ_*i*_ = λ and *α*_*i*_ = *α*. In this case, we can say more about steady-state densities.

**Proposition 7** *Consider a RFMEOD with dimension n and with particle size ℓ and λ_i_ = λ and α_i_ = α (≠0). Then*

$$\begin{array}{c} e_{i}={\left(\frac{\lambda +\alpha }{\lambda }\right)}^{n-i}e_{n},\;\;\text{for}\;\;i=n-\ell +1,\ldots ,n, \end{array}$$ (31)

$$\begin{array}{c} e_{1}>e_{2}>\ldots >e_{n-\ell +1}>e_{n-\ell +2}>\ldots >e_{n}, \end{array}$$ (32)

*and*

$$\begin{array}{c} y_{\ell }>y_{\ell +1}>\cdots >y_{n}. \end{array}$$ (33)

This implies that the steady-state reader densities decrease between sites 1 and *n* and last *ℓ* sites reader density is given by Eq (31). The next example demonstrates this.

**Fig 14:**
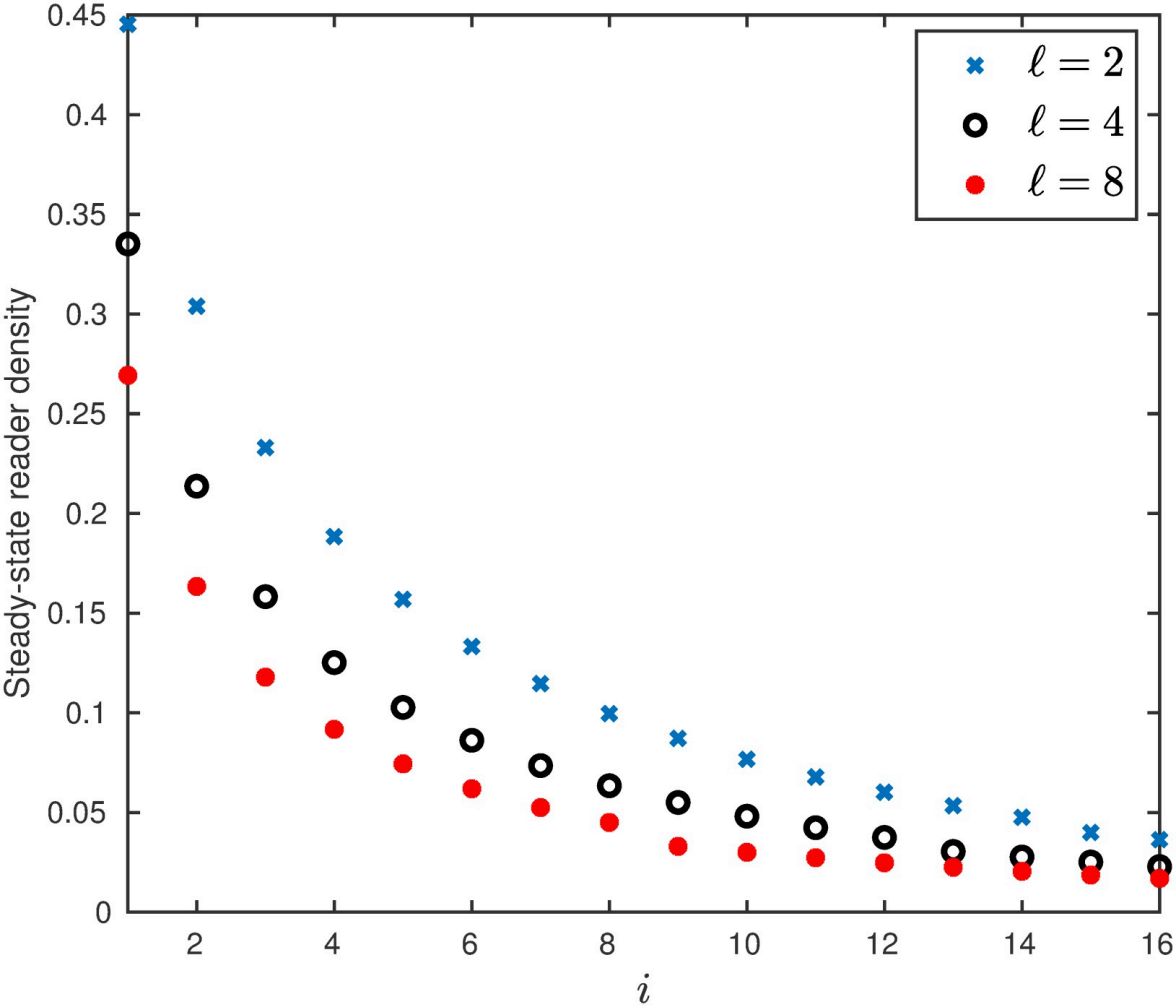
The steady-state reader densities as a function of *i* for a RFMEOD with *n* = 16, λ_0_ = 1, λ_*i*_ = 1, *α*_*i*_ = 0.1, for *i* = 1, 2, …, 16, for different values of *ℓ*.

**Example 12** The steady-state reader densities of the RFMEOD with dimension *n* = 16, for three particle sizes *ℓ* = 2, 4, 8 and λ_0_ = 1, λ_*i*_ = 1 and *α*_*i*_ = 0.1, are depicted in Fig 14. It may be observed that steady-state reader densities monotonically decrease along the mRNA.

It have been seen that for fixed rates, the steady-state protein production rate in the RFMEO with *ℓ* > 1 is always less than the steady-state protein production rate with *ℓ* = 1 [29]. We also observed and investigated through simulations that this seems to hold true even in the case of the presence of drop-off phenomenon. The next example demonstrates that for fixed rates, steady-state output rate in the RFMEOD with *ℓ* > 1 is less than the steady-state output rate in the RFMEOD with *ℓ* = 1.

**Fig 15:**
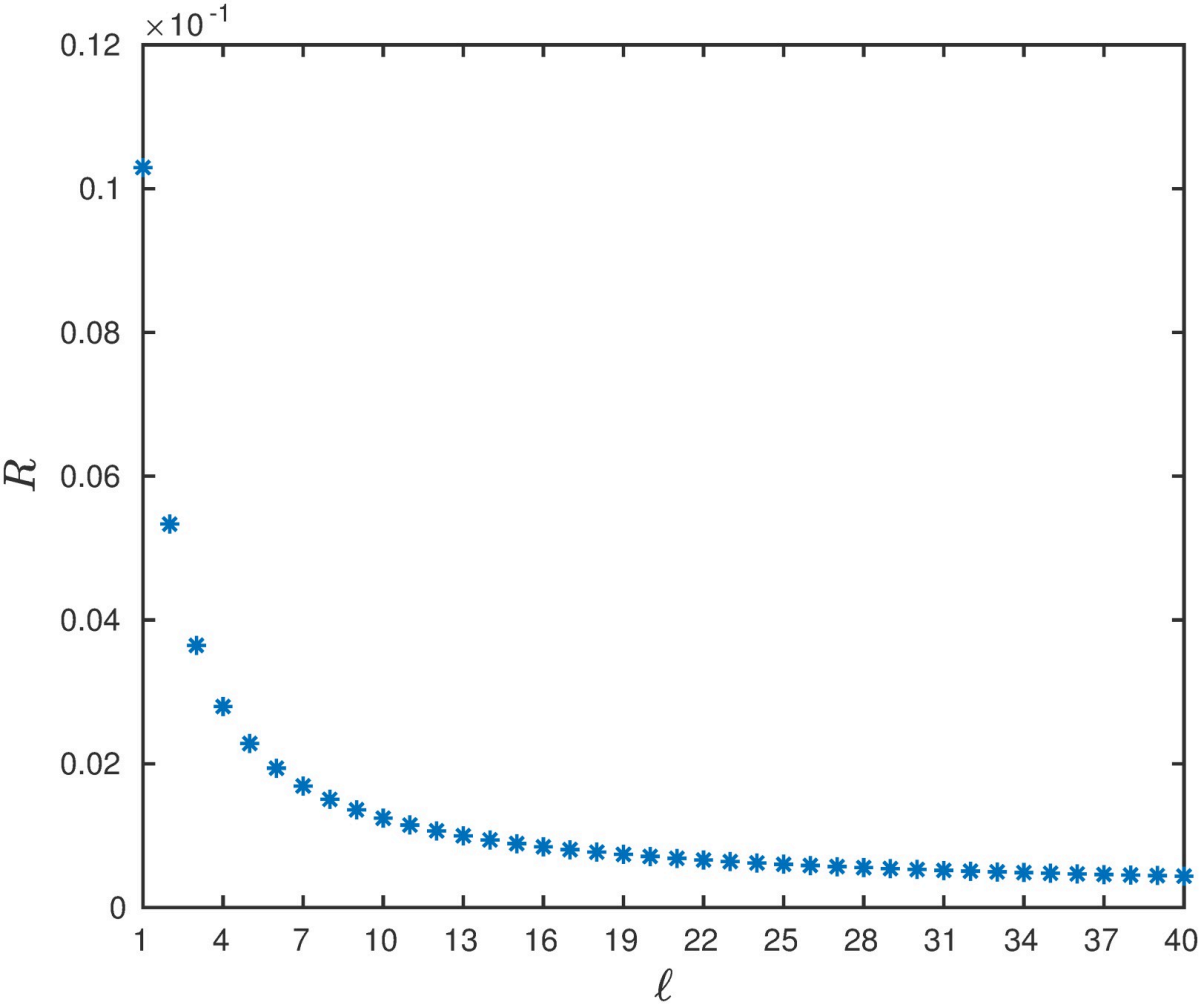
The steady-state output rate *R* as a function of *ℓ* ∈ {1, 2, …, 40} for a RFMEOD with *n* = 300, λ_0_ = 0.8, λ_*i*_ = 1, *α*_*i*_ = 0.01, for all *i*.

**Example 13** Consider a RFMEOD with *n* = 300 sites, ribosome size *ℓ* and rates λ_0_ = 0.8, λ_*i*_ = 1, *α*_*i*_ = 0.01, for all *i*. It can be seen that in Fig 15 that *R* monotonically decreases with *ℓ*.

### 4.2 RFMEOD with positive feedback

In eukaryotes, mRNA molecules sometimes form circular structures which promote recycling of the ribosomal subunits [63–65]. Therefore, it is biologically evident to include the fact that the translation initiation rate is affected by the premature and complete translation termination rate. This model can be used to fine tune the rate of protein production by ribosome-recycling in the case of changing ribosomal availability due to environmental stress [66]. We analyze the behavior of the RFMEOD as a control system after closing the loop from the output of ribosomes to input with positive linear feedback.

Consider the RFMEOD with feedback:

$$\begin{array}{c} \begin{array}{cc} {\dot{x}}_{1} & =\left(k_{1}+k_{2}\left(\lambda_{n}x_{n}+\sum_{i=1}^{n}\alpha_{i}x_{i}\right)\right)\left(1-y_{\ell }\right)-\lambda_{1}x_{1}\left(1-y_{\ell +1}\right)-\alpha_{1}x_{1},\\ {\dot{x}}_{2} & =\lambda_{1}x_{1}\left(1-y_{\ell +1}\right)-\lambda_{2}x_{2}\left(1-y_{\ell +2}\right)-\alpha_{2}x_{2},\\ \vdots \\ {\dot{x}}_{n-\ell +1} & =\lambda_{n-\ell }x_{n-\ell }\left(1-y_{n}\right)-\lambda_{n-\ell +1}x_{n-\ell +1}-\alpha_{n-\ell +1}x_{n-\ell +1},\\ {\dot{x}}_{n-\ell +2} & =\lambda_{n-\ell +1}x_{n-\ell +1}-\lambda_{n-\ell +2}x_{n-\ell +2}-\alpha_{n-\ell +2}x_{n-\ell +2},\\ \vdots \\ {\dot{x}}_{n} & =\lambda_{n-1}x_{n-1}-\lambda_{n}x_{n}-\alpha_{n}x_{n}, \end{array} \end{array}$$ (34)

where *k*_1_ > 0 and *k*_2_ ≥ 0.

**Fig 16:**
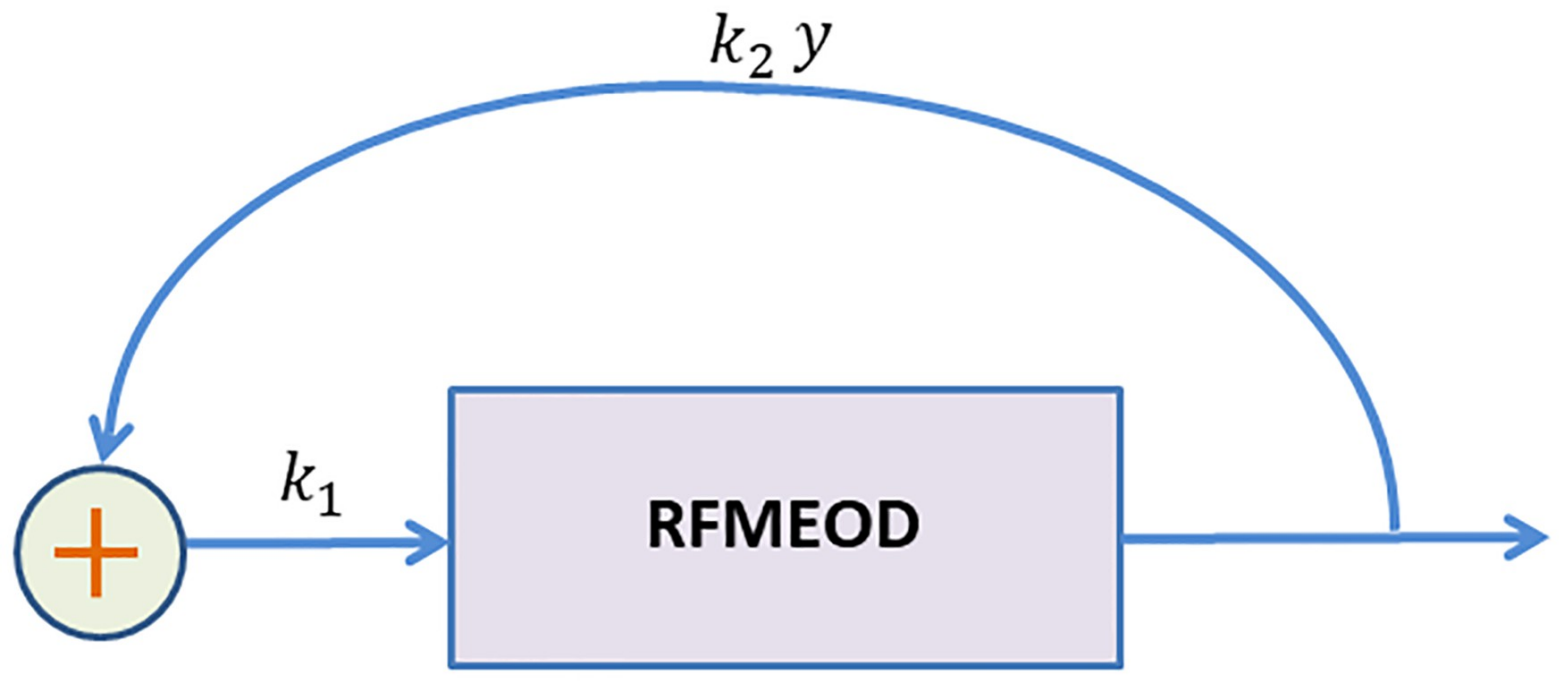
The EFEIOD with feedback where parameters *k*_1_ and *k*_2_ represents the constant source and recycling rate of ribosomes respectively. The term $y=\lambda_{n}x_{n}+\sum_{i=1}^{n}\alpha_{i}x_{i}$ denotes the output of ribosomes from the system.

Here, the parameter *k*_1_ represents the diffusion of ribosomes to the start codon of a mRNA molecule that is not related to recycling of ribosomes. The term $k_{2}\left(\lambda_{n}x_{n}+\sum_{i=1}^{n}\alpha_{i}x_{i}\right)$ represents the feedback due to recycling of ribosomes that have finished (partially or completely) the process of translating the mRNA as depicted in Fig 16.

This is a generalization of the original RFMEOD as it includes both a term related to initiation rate with and without recycling of ribosomes. The next theorem proves that trajectories from any initial condition in *H* will always converge to a unique equilibrium point in *H*.

**Theorem 3** *The set H includes a unique equilibrium steady-state density e of the closed loop system* (34). *This equilibrium point is globally asymptotically stable in H, i.e. lim_t→∞_x(t, a) = e for any initial condition a ∈ H*.

**Fig 17:**
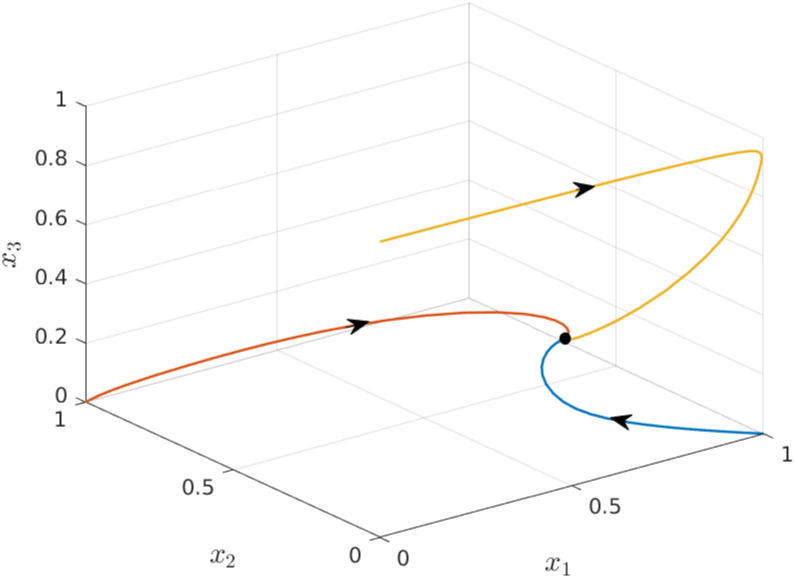
Trajectories of RFMEOD for three initial conditions given in Example 14 as a function of time. The equilibrium point is marked by a ellipse.

**Example 14** Consider the closed loop system (34) with dimension *n* = 3, *ℓ* = 2, λ_*i*_ = 1, *α*_*i*_ = 0.01, *k*_1_ = 1 and *k*_2_ = 100. Fig 17 depicts trajectories for three different initial conditions [1, 0, 0], [0, 1, 0] and [0, 0, 1] in *H*.

The next result provides information on the change of *e* w.r.t. the control parameters *k*_1_ and *k*_2_.

**Proposition 8** *Suppose that the* λ*_i_s and α_i_s are fixed. Let e and* $\bar{e}$ *correspond to the control parameters (k_1_, k_2_) and* $\left({\bar{k}}_{1},{\bar{k}}_{2}\right)$ *respectively. If* $k_{1}={\bar{k}}_{1}$ *then* $e_{n}<{\bar{e}}_{n}$ *if and only if* $k_{2}<{\bar{k}}_{2}$. *If* $k_{2}={\bar{k}}_{2}$ *then* $e_{n}<{\bar{e}}_{n}$ *if and only if* $k_{1}<{\bar{k}}_{1}$.

From a biophysical point of view, the above result inferred the intuitive result that increasing in any of the control parameters leads to an increase in the protein production rate. This result may be useful in context of biotechnology in order to improve levels of proteins in the host.

### 4.3 Validation through Monte Carlo simulations

Since the RFMEOD is a mean-field approximation of TASEP with extended objects and include an additional detachment rate at every site of the lattice, we ran MATLAB simulations of this process. A simulation begins with an empty chain of dimension *n* and continues for 10^8^ time steps i.e. total simulation time. Each site can accommodate atmost one particle and a particle can only hop unidirectionally to a consecutive site if it is empty. The leftmost site that particle is covering is referred to as the reader. Every site *i*, *i* = 1, 2, …, *n* in the chain is associated with hopping rates λ_*i*_s and detachment rates *α*_*i*_s where the next hopping event time *t*_*k*_ + *ϵ*_*k*_ or the next detachment event time *t*_*k*_ + *δ*_*k*_ is generated randomly. For site *i*, *ϵ*_*k*_ and *δ*_*k*_ are random variables drawn from the exponential distribution with mean rate λ_*i*_ and *α*_*i*_ respectively. If hopping time is equal to the simulation time, then the reader at site *i* hops to site *i* + 1, provided site *i* + *ℓ* is empty. Similarly, if the detachment event time is equal to the simulation time then the reader dissociates from site *i*. The occupancy at each site is averaged throughout the simulations with the first 10^6^ time steps discarded from the calculations to obtain the average steady-state reader density of each site.

In the example below, we show that simulations supports the modeling of dynamical aspects of translation using RFMEOD.

**Fig 18:**
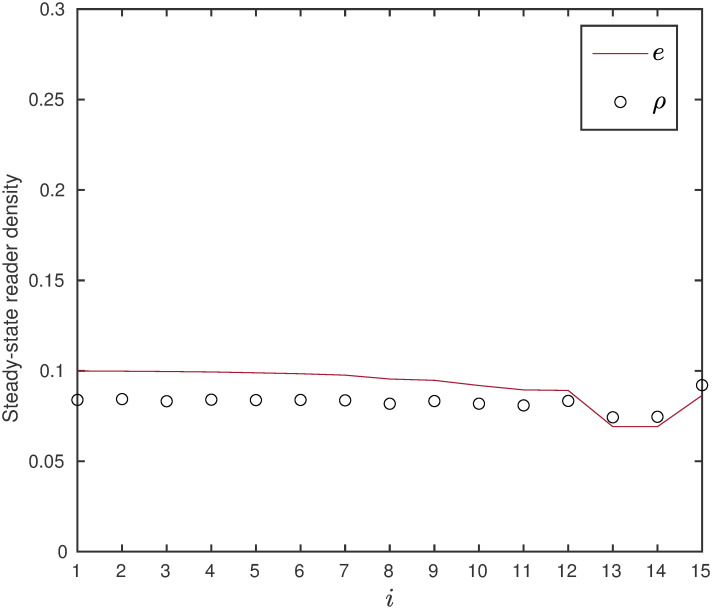
Steady-state reader density as a function of site number *i* given in Example 15.

**Example 15** Consider the RFMEOD with dimension *n* = 15, particle size *ℓ* = 3, rates λ_0_ = 0.1, λ_*i*_ = 1, for *i* = 1, 2, …, *n* − *1*, λ_*n*_ = 0.8 and *α*_*i*_ = 0 except for *α*_8_ = 0.01. Fig 18 depicts steady-state reader density *e* and *ρ* for RFMEOD and TASEP-detachment respectively.

## 5 Discussion

In many biological processes like translation, cellular transport, gene transcription and many more, ‘particles’ move along one-dimensional “tracks”. We studied a deterministic model called EFEIOD for the flow of particles along an ordered lattice of sites that encapsulates important cellular properties like detachment of particles from any site, nearest-neighbor interactions and the fact that most particles cover more than one site along the lattice. We analyzed this model using tools from systems and control theory, in particular contraction theory.

We proved that the EFEIOD converges to a unique steady-state density for any set of feasible parameters. In other words, EFEIOD is robust to the initial conditions. Moreover, we prove that if one or more of the parameters are time-varying periodic functions with a common period *T*, then the steady-state densities also converge to a periodic solution with period *T*. We demonstrate through simulations of the EFEIOD several useful observations. For example, increasing the particle size may sometimes lead to an increase in the output rate in the presence of weak repulsions. Surprisingly, we also show that increasing the detachment rate does not always decrease the output rate as elucidated in [28]. It is also important to note that several known models like RFM with positive feedback [19], RFMEO [29] and the model used in [67] for mRNA translation are special cases of the proposed model.

We also rigorously analyze a special case of the EFEIOD when *q* = *r* = 1 and called it RFMEOD to analyze the effects of ribosome drop-off on the translation process. The ribosome drop-off is important to study as it could significantly deteriorate the fitness of the host. We proved that increasing any one of the transition (detachment) rates of the RFMEOD always increases (decreases) the steady-state protein production and that in the homogeneous case, i.e. when all the transition rates are equal and all the detachment rates are equal, the reader density monotonically decreases along the lattice. We also modeled the observed phenomenon that many eukaryotic ribosomes may translate mRNA in multiples by including positive linear feedback in the RFMEOD.

The results reported can shed light on many biophysical properties of intracellular transport and may prove useful for applications in synthetic biology. One may consider to integrate another realistic feature of the cellular transport such as attachment of biological particles at different sites along the tracks in our model. Another research topic is studying the networks of EFEIOD and considering various phenomena like competition of resources in the network. We believe that the EFEIOD can be generalized to model and analyze more natural and artificial processes. Examples include coordination of large groups of organisms, traffic control and more.

## 6 Appendix

*Proof of Proposition 1 and 2*: The fact that *H* is an invariant set of the dynamics and have a repelling boundary follows from the equations from the EFEIOD. Let

$$\begin{array}{c} \eta_{i}\left(t\right)≔\lambda_{i}\left(1+\left(q-1\right)z_{i+\ell +1}\right)\left(1+\left(r-1\right)z_{i-\ell }\right),\;i=0,1,\ldots ,n \end{array}$$ (35)

and

$$\begin{array}{c} \delta_{i}\left(t\right)≔\alpha_{i}\left(1+\left(r-1\right)z_{i+\ell }\right)\left(1+\left(r-1\right)z_{i-\ell }\right) \end{array}$$ (36)

with the *z*_*i*_*s* defined in Eq (2). Therefore, EFEIOD can be written as

$$\begin{array}{c} {\dot{x}}_{1}=\eta_{0}\left(t\right)\;\left(1-w_{\ell }\right)-\eta_{1}\left(t\right)\;x_{1}\left(t\right)\;\left(1-w_{\ell +1}\right)-\delta_{1}\;x_{1} \end{array}$$ (37)

and

$$\begin{array}{c} {\dot{x}}_{i}=\eta_{i-1}\left(t\right)\;x_{i-1}\left(t\right)\;\left(1-w_{i+\ell -1}\right)-\eta_{i}\left(t\right)\;x_{i}\left(t\right)\;\left(1-w_{i+\ell }\right)-\delta_{i}\;x_{i}\;i=2,3,\ldots ,n. \end{array}$$ (38)

with the *w*_*i*_*s* defined in Eq (3).

Note that for *r*, *q* > 0 all the time-varying transition rates *η*_*i*_(*t*) are uniformly separated from zero and uniformly bounded and all the time-varying detachment rates are non-negative and uniformly bounded. Now, the proof of proposition follows from the results in [29, 68].

*Proof of Proposition 3*: Let,

$$\begin{array}{c} \psi_{i}\left(t\right)≔\eta_{i}\left(t\right)\;\frac{\left(1-w_{i+\ell }\left(t\right)\right)}{\left(1-x_{i+1}\left(t\right)\right)},\;i=0,1,\ldots ,n-1 \end{array}$$ (39)

and

$$\begin{array}{c} \psi_{n}\left(t\right)≔\eta_{n}\left(t\right)\;\left(1-w_{n+\ell }\left(t\right)\right). \end{array}$$ (40)

Now, combining the representations in Eqs 39 and 40 with the Eqs 37 and 38, we get

$$\begin{array}{c} {\dot{x}}_{1}=\psi_{0}\;\left(1-x_{1}\right)-\psi_{1}\;x_{1}\left(1-x_{2}\right)-\delta_{1}\;x_{1}, \end{array}$$ (41)

$$\begin{array}{c} {\dot{x}}_{i}=\psi_{i-1}\;x_{i-1}\left(1-x_{i}\right)-\psi_{i}\;x_{i}\left(1-x_{i+1}\right)-\delta_{i}\;x_{i},\;i=2,\ldots ,n-1, \end{array}$$ (42)

$$\begin{array}{c} {\dot{x}}_{n}=\psi_{n-1}\;x_{n-1}\left(1-x_{n}\right)-\psi_{n}\;x_{n}-\delta_{n}\;x_{n}. \end{array}$$ (43)

Proposition 1 and the equations above imply that that EFEIOD can be interpreted as time-varying MFALK system with no backward and attachment dynamics with the well defined rates for all *t* > 0. Write the time-varying MFALK as $\dot{x}=f\left(x,t\right)$ with transition rates *ψ*_*i*_(*t*) and detachment rates *δ*_*i*_(*t*). A calculation shows that the Jacobian of *f* is *J*(*t*, *x*) = *L*(*t*, *x*) + *D*(*t*), where *L* is the matrix,

$$J_{1}=[\begin{array}{ccccc} -\psi_{1}\left(1-x_{2}\right) & \psi_{1}x_{1} & 0 & \ldots & 0\\ \psi_{1}\left(1-x_{2}\right) & -\psi_{1}x_{1}-\psi_{2}\left(1-x_{3}\right) & \psi_{2}x_{2} & \ldots & 0\\ 0 & \psi_{2}\left(1-x_{3}\right) & -\psi_{2}x_{2}-\psi_{3}\left(1-x_{4}\right) & \ldots & 0\\ & & \vdots \\ 0 & 0 & \ldots & \psi_{n-1}\left(1-x_{n}\right) & -\psi_{n-1}x_{n-1} \end{array}]$$

and *D* is the diagonal matrix

$$\begin{array}{c} D=diag(-\psi_{0}-\delta_{1},-\delta_{2},\ldots ,-\delta_{n-1},-\psi_{n}-\delta_{n}). \end{array}$$ (44)

Hence Proposition 2 and the results in [21, 68] imply that the EFEIOD is SOST on *H* and this completes the proof.

*Proof of Proposition 4*: It follows from the results in [21] and the argument used in the proof of proposition 3 in [23].

*Proof of Proposition 5*: Consider two RFMEODs both with same dimension *n*, particle size *ℓ*, rates λ_*i*_ for all *i* = 0, 1, …, *n* and *α*_*i*_ for *i* = 1, 2, …, *n* except for any one *j* ∈ {1, 2, …, *n* − 1} such that

$$\begin{array}{c} \alpha_{j}<{\bar{\alpha }}_{j}. \end{array}$$ (45)

Therefore, the first RFMEOD admits a steady-state production rate *R* and the second one admits $\bar{R}$. We have to prove that $\bar{R}<R$. We shall prove it by contradiction.

Let us assume that

$$\begin{array}{c} R\leq \bar{R} \end{array}$$ (46)

which implies that from equation

$$\begin{array}{c} e_{n}\leq {\bar{e}}_{n}. \end{array}$$ (47)

From Eqs (46), (47) and (26), we have

$$\begin{array}{c} e_{n-1}\leq {\bar{e}}_{n-1}. \end{array}$$ (48)

We start with the case *j* = *n* − 1. From Eqs (26) and (46)

$$\begin{array}{c} \lambda_{n-2}\;e_{n-2}-\alpha_{n-1}\;e_{n-1}\leq \lambda_{n-2}\;{\bar{e}}_{n-2}-{\bar{\alpha }}_{n-1}\;{\bar{e}}_{n-1}. \end{array}$$ (49)

Now, Eqs (45) and (49) implies that

$$\begin{array}{c} \lambda_{n-2}\;e_{n-2}-{\bar{\alpha }}_{n-1}\;e_{n-1}\leq \lambda_{n-2}\;{\bar{e}}_{n-2}-{\bar{\alpha }}_{n-1}\;{\bar{e}}_{n-1} \end{array}$$ (50)

which implies that

$$\begin{array}{c} e_{n-2}<{\bar{e}}_{n-2}. \end{array}$$ (51)

Continuing this way, we have

$$\begin{array}{c} e_{j}\leq {\bar{e}}_{j}\;\text{for}\;j=n-\ell +1,\cdots ,n-2. \end{array}$$ (52)

This means that

$$\begin{array}{c} y_{n}<{\bar{y}}_{n}. \end{array}$$ (53)

Now, from Eqs (26), (45) and (46), we have

$$\begin{array}{c} \lambda_{n-\ell }\;e_{n-\ell }\;\left(1-y_{n}\right)-\sum_{i=n-\ell +1}^{n-1}\alpha_{i}\;e_{i}\leq \lambda_{n-\ell }\;{\bar{e}}_{n-\ell }\;\left(1-{\bar{y}}_{n}\right)-\sum_{i=n-\ell +1}^{n-1}\alpha_{i}\;{\bar{e}}_{i}. \end{array}$$ (54)

Combining this with Eqs (48), (52) and (53), we have

$$\begin{array}{c} e_{n-\ell }<{\bar{e}}_{n-\ell }. \end{array}$$ (55)

Continuing this way, we have

$$\begin{array}{c} e_{j}<{\bar{e}}_{j}\;\text{for}\;j=1,\cdots ,n-2. \end{array}$$ (56)

which implies that

$$\begin{array}{c} y_{\ell }<{\bar{y}}_{\ell }. \end{array}$$ (57)

From Eqs (26) and (45), consider

$$\begin{array}{c} \lambda_{0}\;\left(1-y_{\ell }\right)-\sum_{i=1}^{n-1}\alpha_{i}\;e_{i}\leq \lambda_{0}\;\left(1-{\bar{y}}_{\ell }\right)-\sum_{i=1}^{n-1}\alpha_{i}\;{\bar{e}}_{i}. \end{array}$$ (58)

From Eqs (47) and (58) we have

$$\begin{array}{c} y_{\ell }\geq {\bar{y}}_{\ell }. \end{array}$$ (59)

which is the contradiction to Eq (57) resulting in $\bar{R}<R$ in the case $\alpha_{n-1}<{\bar{\alpha }}_{n-1}$.

Hence, using the same approach for any *j* ∈ {1, 2, …, *n* − 1}, we can conclude that $\bar{R}<R$.

*Proof of Proposition 6*: The proof is similar to the proof of proposition 4 above and is thus omitted.

*Proof of Proposition 7*: From Eq (26), we have

$$\begin{array}{c} \lambda \;e_{n-1}=\left(\lambda +\alpha \right)\;e_{n}\Rightarrow e_{n-1}=(\frac{\lambda +\alpha }{\lambda })\;e_{n}. \end{array}$$ (60)

Similarly, we have

$$\begin{array}{c} e_{j}={\left(\frac{\lambda +\alpha }{\lambda }\right)}^{n-j}\;e_{n}\;\;\text{for}\;\;j=n-\ell +1,\cdots ,n. \end{array}$$ (61)

Since (λ + *α*) > λ, we have

$$\begin{array}{c} e_{n-\ell +1}>e_{n-\ell +2}>\cdots >e_{n}. \end{array}$$ (62)

From Eq (26), consider

$$\begin{array}{c} \lambda \;e_{n-\ell }\;\left(1-y_{n}\right)-\sum_{i=n-\ell +1}^{n-1}\alpha \;e_{i}=\lambda \;e_{n-\ell +1}-\sum_{i=n-\ell +2}^{n-1}\alpha \;e_{i} \end{array}$$ (63)

which implies

$$\begin{array}{c} \lambda \;e_{n-\ell }\;\left(1-y_{n}\right)=\lambda \;e_{n-\ell +1}+\alpha \;e_{n-\ell +1}. \end{array}$$ (64)

Therefore,

$$\begin{array}{c} e_{n-\ell }\;\left(1-y_{n}\right)>e_{n-\ell +1}\Rightarrow e_{n-\ell }>e_{n-\ell +1}. \end{array}$$ (65)

From Eq (62),

$$\begin{array}{c} e_{n-\ell }>e_{n-\ell +1}>e_{n-\ell +2}>\cdots >e_{n}. \end{array}$$ (66)

Now, consider

$$\begin{array}{c} y_{n-1}-y_{n}=e_{n-\ell }-e_{n}>0\Rightarrow y_{n-1}>y_{n}. \end{array}$$ (67)

Now, from Eq (26),

$$\begin{array}{c} \lambda \;e_{n-\ell -1}\;\left(1-y_{n-1}\right)=\lambda \;e_{n-\ell }\;\left(1-y_{n}\right)+\alpha \;e_{n-\ell } \end{array}$$ (68)

which implies

$$\begin{array}{c} e_{n-\ell -1}\;\left(1-y_{n-1}\right)>e_{n-\ell }\;\left(1-y_{n}\right). \end{array}$$ (69)

From Eq (67), we have

$$\begin{array}{c} e_{n-\ell -1}>e_{n-\ell } \end{array}$$ (70)

and thus

$$\begin{array}{c} y_{n-2}>y_{n-1}. \end{array}$$ (71)

Continuing in this way completes the proof.

*Proof of Theorem 3*: Clearly, *H* is an invariant set of the dynamics. Note that this system is RFMEOD with time-varying initiation rate which is uniformly bounded and uniformly separated from zero i.e.

$$\begin{array}{c} 0<k_{1}+k_{2}\left(\lambda_{n}x_{n}+\sum_{i=1}^{n}\alpha_{i}x_{i}\right)<M. \end{array}$$ (72)

Now the proof follows by theorem 1.

*Proof of Proposition 8*: We have equations for the RFMEOD with feedback at steady-state as follows:

$$\begin{array}{cc} e_{n} & ≔\frac{R}{\lambda_{n}},\\ e_{i} & ≔\frac{R+\sum_{k=i+1}^{n-1}g_{k}\left(e_{k}\right)}{\lambda_{i}\left(1-y_{i+\ell }\right)},\;i=n-1,\cdots ,1,\\ \text{and}\;\text{also}\\ y_{\ell } & ≔1-\frac{R+\sum_{k=1}^{n-1}g_{k}\left(e_{k}\right)}{\lambda_{0}\;\left(k_{1}+k_{2}\left(\lambda_{n}e_{n}+\sum_{i=1}^{n}\alpha_{i}e_{i}\right)\right)}. \end{array}$$ (73)

Suppose that $k_{1}={\bar{k}}_{1}$ and $k_{2}<{\bar{k}}_{2}$. We have to prove that $e_{n}<{\bar{e}}_{n}$. We shall prove it by contradiction. Assume

$$\begin{array}{c} {\bar{e}}_{n}\leq e_{n} \end{array}$$ (74)

which implies that

$$\begin{array}{c} \bar{R}\leq R. \end{array}$$ (75)

which further implies from Eq (73) that

$$\begin{array}{c} {\bar{e}}_{i}\leq e_{i}\;\text{for}\;\text{all}\;i=1,2,\cdots ,n-1. \end{array}$$ (76)

Therefore,

$$\begin{array}{c} {\bar{y}}_{\ell }\leq y_{\ell }. \end{array}$$ (77)

From Eq (73) and simplifying calculations we have,

$$\begin{array}{c} y_{\ell }-{\bar{y}}_{\ell }=k_{1}\;\left(\lambda_{n}+\alpha_{n}\right)\;\left(\bar{e_{n}}-e_{n}\right)+\left(k_{2}-{\bar{k}}_{2}\right)\;\left(\lambda_{n}\;\left(\lambda_{n}+\alpha_{n}\right)\;{\bar{e}}_{n}+\alpha_{n}\;\left(\lambda_{n}+\alpha_{n}\right)\;e_{n}\;{\bar{e}}_{n}\right)\\ \;+\left(k_{2}-{\bar{k}}_{2}\right)\;(\left(\lambda_{n}+\alpha_{n}\right)\;\sum_{i=1}^{n-1}\left({\bar{e}}_{i}\;e_{n}+e_{i}\;{\bar{e}}_{n}\right)+(\sum_{i=1}^{n-1}\alpha_{i}\;e_{i})\;(\sum_{i=1}^{n-1}\alpha_{i}\;{\bar{e}}_{i})). \end{array}$$ (78)

The fact that $k_{2}<{\bar{k}}_{2}$ and Eqs (74) and (78) implies that

$$\begin{array}{c} y_{\ell }<{\bar{y}}_{\ell } \end{array}$$ (79)

which is a contradiction to Eq (77) and hence $e_{n}<{\bar{e}}_{n}$ and the other part follows the same arguments.
